# Cationic Ring‐Opening Polymerization‐Induced Self‐Assembly (CROPISA) of 2‐Oxazolines: From Block Copolymers to One‐Step Gradient Copolymer Nanoparticles

**DOI:** 10.1002/anie.202416106

**Published:** 2024-12-09

**Authors:** Niccolò Lusiani, Ewa Pavlova, Richard Hoogenboom, Ondrej Sedlacek

**Affiliations:** ^1^ Department of Physical and Macromolecular Chemistry, Faculty of Science Charles University 128 40 Prague 2 Czech Republic; ^2^ Institute of Macromolecular Chemistry, v.v.i Academy of Sciences of the Czech Republic Heyrovsky Sq. 2 162 06 Prague 6 Czech Republic; ^3^ Department of Organic and Macromolecular Chemistry, Supramolecular Chemistry Group, Centre of Macromolecular Chemistry (CMaC), Faculty of Sciences Ghent University Krijgslaan 281 S4 9000 Ghent Belgium

**Keywords:** Copolymerization, CROP, Nanoparticles, PISA, Poly(2-oxazoline)s

## Abstract

In recent years, polymerization‐induced self‐assembly (PISA) has emerged as a powerful method for the straightforward synthesis of polymer nanoparticles at high concentration. In this study, we describe for the first time the synthesis of poly(2‐oxazoline) nanoparticles by dispersion cationic ring‐opening polymerization‐induced self‐assembly (CROPISA) in *n*‐dodecane. Specifically, a *n*‐dodecane‐soluble aliphatic poly(2‐(3‐ethylheptyl)‐2‐oxazoline) (PEHOx) block was chain‐extended with poly(2‐phenyl‐2‐oxazoline) (PPhOx). While the PhOx monomer is soluble in *n*‐dodecane, its polymerization leads to *n*‐dodecane‐insoluble PPhOx, which leads to in situ self‐assembly of the formed PEHOx‐*b*‐PPhOx copolymers. The polymerization kinetics and micellization upon second block formation were studied, and diverse nanoparticle dispersions were prepared, featuring varying block lengths and polymer concentrations, leading to dispersions with distinctive morphologies and physical properties. Finally, we developed a single‐step protocol for the synthesis of polymer nanoparticles directly from monomers via gradient copolymerization CROPISA, which exploits the significantly greater reactivity of EHOx compared to that of PhOx during the statistical copolymerization of both monomers. Notably, this approach provides access to formulations with monomer compositions otherwise unattainable through the block copolymerization method. Given the synthetic versatility and application potential of poly(2‐oxazolines), the developed CROPISA method can pave the way for advanced nanomaterials with favorable properties as demonstrated by using the obtained nanoparticles for stabilization of Pickering emulsions.

## Graphical abstract

This study introduces a novel method for creating poly(2‐oxazoline) nanoparticles via cationic ring‐opening polymerization‐induced self‐assembly (CROPISA) in *n*‐dodecane. By chain‐extending poly(2‐(3‐ethylheptyl)‐2‐oxazoline) with poly(2‐phenyl‐2‐oxazoline), unique nanoparticles with diverse morphologies were synthesized. Additionally, a single‐step gradient CROPISA process was developed, enabling a streamlined synthesis of new formulations.

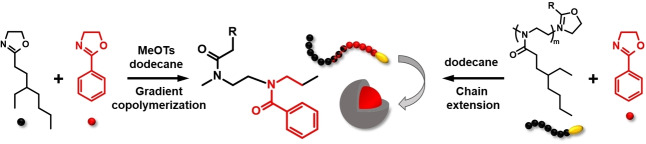

## Introduction

Polymerization‐induced self‐assembly (PISA) has been recognized for nearly a decade as an innovative and versatile tool in the fields of materials science and nanotechnology.[^1^, ^2^, ^3^, ^4^] This technique combines controlled polymerization with self‐assembly to create a wide range of well‐defined nanostructured materials. Generally, PISA proceeds via chain extension of a solvophilic polymer block with a second block that becomes solvophobic upon polymerization of a soluble monomer.[^5^, ^6^, ^7^] In this way, amphiphilic block copolymers are synthesized, leading to the in situ formation of self‐assembled dispersions with potentially high colloidal concentrations and with precise control over size, shape, and composition during polymerization.^[7]^ Alternatively, with the statistical copolymerization of a faster‐reacting solvophilic monomer and a slower reacting solvophobic monomer, it is possible to synthesize gradient copolymer nanoparticles by PISA via a one‐step approach.^[8]^ The use of gradient PISA (gPISA) has not been widely reported because of the additional constraint related to the reactivity ratios of the two monomers. Nonetheless, higher‐order worm‐like morphologies have been obtained by Boyer and coworkers via the statistical copolymerization of oligo(ethylene glycol) methyl ether methacrylate (OEGMA) with diacetone acrylamide in water.^[9]^

While PISA has been extensively reported in water[^10^, ^11^, ^12^] and water‐miscible solvents,^[7]^ more recent reports have demonstrated the potential of PISA in non‐polar solvents, such as heptane, *n*‐dodecane, or mineral oils, with potential applications of such nanoparticle formulations as low‐viscosity lubricants,[^7^, ^13^] and Pickering emulsifiers^][14]^ PISA is commonly performed via controlled radical polymerizations, particularly via reversible addition−fragmentation transfer (RAFT) polymerization,[^5^, ^6^, ^7^, ^10^, ^12^, ^15^, ^16^] while we are aware of only a few reports on PISA via ionic polymerization using living anionic polymerization.[^17^, ^18^, ^19^] To the best of our knowledge, cationic polymerization has never been reported for PISA. Although cationic polymerizations require strict operating conditions and can impose constraints on the applicable monomers and the compatibility of the polymerization solvent, it typically proceeds in a living and controlled manner. Finally, cationic polymerization can be utilized for the synthesis of polymers not attainable by other polymerization techniques, such as poly(2‐alkyl/aryl‐2‐oxazoline)s (PAOx).

PAOx have become an intensively investigated class of polymers because of their structural tunability, versatility, and biocompatibility.[^20^, ^21^, ^22^, ^23^, ^24^, ^25^] Generally, PAOx are synthesized by cationic ring‐opening polymerization (CROP) of 2‐oxazoline monomers initiated by electrophiles (e. g., alkyl halides, triflates or tosylates),[^26^, ^27^, ^28^] which leads to stable tertiary amide‐containing polymers. Owing to their favorable biological properties, PAOx currently represent one of the leading alternative polymer platforms to the widely used immunogenic polyethylene glycol in biomedical applications.[^29^, ^30^, ^31^] Furthermore, PAOx have found their applications in different areas, such as antifouling coatings,^[32]^ stabilizers for emulsion polymerization,^[33]^ and micellar catalysis.^[34]^

In particular, PAOx represent an appealing platform for the synthesis of self‐assembled block or gradient copolymer nanoparticles.[^34^, ^35^, ^36^, ^37^, ^38^, ^39^] For example, Luxenhofer, et al., reported extremely high loadings of hydrophobic anticancer drugs in self‐assembled block copolymer PAOx nanoparticles, with potential applications in anticancer therapy. Furthermore, CROP is particularly suitable for the controlled statistical copolymerization of 2‐oxazoline monomers with different reactivities and solvophilicities, leading to amphiphilic gradient copolymers formed directly from the statistical copolymerization of the monomers in a single step.[^40^, ^41^, ^42^] Amphiphilic gradient copolymers can then self‐assemble into nanoparticles in a similar fashion as their block copolymer analogs.

The synthesis of PAOx nanoparticles via the PISA approach represents an attractive research goal, but thus far, it has never been reported because of the aforementioned restrictions imposed by cationic polymerization. First, CROPISA cannot be performed in water, alcohol, or other protic solvents that interfere with the polymerization process. Furthermore, PISA requires a solvent that selectively solvates only one of the PAOx blocks, which is not achievable for the most commonly employed solvents for the CROP of PAOx, such as acetonitrile, benzonitrile, and sulfolane.[^43^, ^44^, ^45^, ^46^] In most cases, these polar aprotic solvents solubilize all commonly studied PAOx irrespective of their structure.

In this report, we propose *n*‐dodecane as a selective solvent for the CROPISA of 2‐oxazolines. Specifically, *n*‐dodecane is an excellent solvent for aliphatic side‐chain poly(2‐(3‐ethylheptyl)‐2‐oxazoline) (PEHOx), in which the branching suppresses crystallization, while being a poor solvent for aromatic poly(2‐phenyl‐2‐oxazoline (PPhOx).^[47]^ Therefore, we studied the chain extension of in situ‐formed PEHOx blocks with 2‐phenyl‐2‐oxazoline (PhOx) to achieve the in situ self‐assembly of PEHOx‐*b*‐PPhOx nanoparticles in *n*‐dodecane via CROPISA. After the polymerization kinetics study, a series of block copolymer nanoparticles were synthesized with varying lengths of both blocks, as well as varying monomer/polymer concentrations. The nanoparticles were characterized by DLS, TEM and SAXS to describe the impact of the copolymer structure on the nanoparticle size and morphology. Finally, we developed a one‐step protocol for the direct synthesis of gradient PEHOx‐*g*‐PPhOx nanoparticles via the statistical copolymerization of both monomers in *n*‐dodecane, exploiting the lower reactivity of the PhOx monomer.

## Results and Discussion

First, we screened the solubility of different PAOx in *n*‐dodecane to identify PAOx structures suitable for CROPISA. Initially, 2‐nonyl‐2‐oxazoline was selected as the solvophilic block, but it was not suitable for CROPISA because of its high degree of crystallinity and high melting temperature (150 °C),[^48^, ^49^] which led to its bulk crystallization in *n*‐dodecane during polymerization. Therefore, we employed 2‐(3‐ethylheptyl)‐2‐oxazoline (EHOx), whose branched side chain suppressed the crystallization of the corresponding homopolymer (PEHOx).[^50^, ^51^] Furthermore, we selected poly(2‐phenyl‐2‐oxazoline) (PPhOx) as a solvophobic block, as PPhOx is *n*‐dodecane‐insoluble while the PhOx monomer is soluble at the polymerization temperature of 140 °C. This was confirmed by the solution homopolymerization of PhOx in *n*‐dodecane, which led to the precipitation of the formed PPhOx from the initially homogeneous polymerization mixture (Figure S1).

**Scheme 1:**
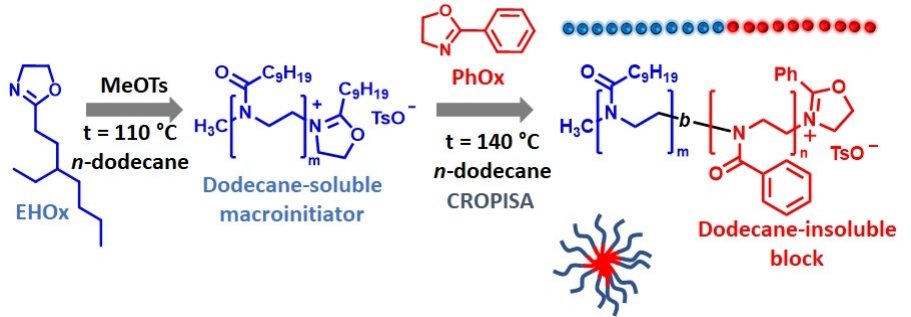
Schematic illustration of the CROPISA of PEHOx_m_‐*b*‐PPhOx_n_ block copolymer nanoparticles in *n*‐dodecane via a one‐pot protocol.

The CROPISA of the nanoparticles was performed by chain‐extending the PEHOx with a PPhOx block in *n*‐dodecane (Scheme 1). CROPISA was performed in a one‐pot two‐step procedure, where EHOx was polymerized in *n*‐dodecane, initiated by methyl *p*‐toluenesulfonate. Upon complete polymerization of the first PEHOx block, the PhOx monomer was added, and the second block was polymerized at 140 °C. The formation of a colloidal dispersion was observed during polymerization, providing the first evidence of successful CROPISA. In general, the dispersions were colloidally stable upon storage at room temperature for at least several weeks.

**Figure 1:**
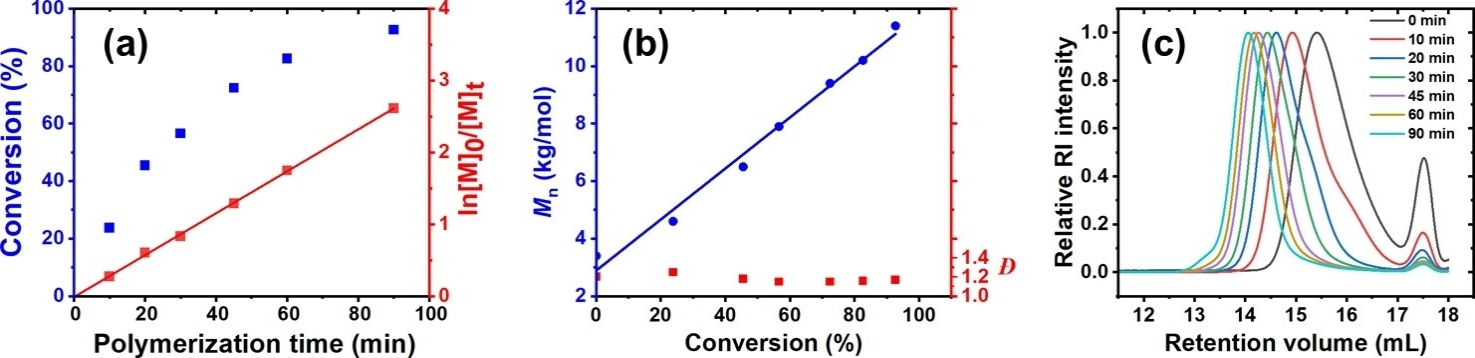
CROPISA kinetics of PhOx initiated by PEHOx living chains in *n*‐dodecane at 140 °C (target DP2 = 60). (a) Evolution of PhOx conversion with reaction time. (b) Evolution of the number‐average molecular weight (Mn) and dispersity (Đ) with PhOx conversion. The linear increase in Mn and the relatively low Đ underscores the lack of significant chain‐transfer reactions. (c) Evolution of the SEC traces in DMA. The peak observed around 17.5 mL in the chromatograms corresponds to lithium tosylate salt.

To further understand the CROPISA process, we investigated the polymerization kinetics of core‐forming block formation in *n*‐dodecane via gas chromatography (GC), size exclusion chromatography (SEC) (Figure 1C), and dynamic light scattering (DLS). The first PEHOx block was synthesized in *n*‐dodecane with the targeted DP_1_ of 18, whereas the length of the second PPhOx block was targeted to be DP_2_ = 60. The consumption of PhOx over time was monitored by GC (Figure 1A). The linear fit of the dependence of the [PhOx]_0_/[PhOx]_t_ logarithm on the polymerization time revealed that the polymerization proceeded via pseudo‐first‐order kinetics with respect to the monomer. Moreover, the linear increase in the number‐average molecular weight (*M*_n_) with monomer conversion, together with the narrow dispersity of the copolymer samples (*Đ* ≤ 1.25 for all samples), indicated that the polymerization was living and proceeding in a controlled manner (Figure 1B).

Nonetheless, the linear pseudo‐first‐order polymerization kinetics of PhOx was unexpected to a certain extent since the polymerization kinetics of the second block in PISA generally follow a sigmoidal curve, with significant acceleration after an induction period.[^52^, ^53^, ^54^, ^55^] As far as it is currently understood, the polymerization rate of the core‐forming monomer is significantly accelerated upon nucleation in the self‐assembly process because of the increased concentration of the monomer in the micellar core in proximity to the reactive chain ends. The different behaviors observed in our system were resolved by DLS investigation of the kinetic samples. Despite the PEHOx living chains being present in *n*‐dodecane as unimers, self‐assembly directly occurred at the beginning of the PhOx polymerization, as the DLS measurements revealed the presence of nanoparticles with *D*_h_=17 nm (Figure S2), which was well above the D_h_ observed for single random coils with similar DPs (~4 nm). This observation highlights that the micellization takes place almost immediately following the addition of very few solvophobic units to the first block. Therefore, the induction time was almost absent, and the entire kinetics process could be described just by the linear accelerated region of the plot. The propagation constant (*k*_p_) derived from the linear fitting was 35×10^−3^ M^−1^ s^−1^, which is similar to that reported for the homopolymerization of PhOx under the same conditions in acetonitrile (*k*_p_=32×10^−3^ M^−1^ s^−1^)^[46]^ and nitromethane (*k*_p_=36×10^−3^ M^−1^ s^−1^).^[56]^ The higher polarity of these solvents typically results in faster propagation rates of CROP than in non‐polar solvents, like *n*‐dodecane,^[57]^ suggesting that monomer partitioning took place leading to faster polymerization of the second block.

In the next step, we used CROPISA to synthesize a library of block copolymer nanoparticles to screen the effects of the copolymer composition on the nanoparticle size and morphology. First, we varied the degree of polymerization of the first block (DP_1_ =15–44) and the second block (DP_2_ =10–210) while maintaining the total solids concentration constant at 25 wt%. The main objective of this part of the research was to explore the range of nanoparticle morphologies and dimensions achievable by CROPISA to generate a phase diagram. Phase diagrams represent an essential tool employed in PISA to reliably reproduce polymer dispersions with the desired properties by selecting the correct polymerization parameters.[^58^, ^59^]

**Figure 2:**
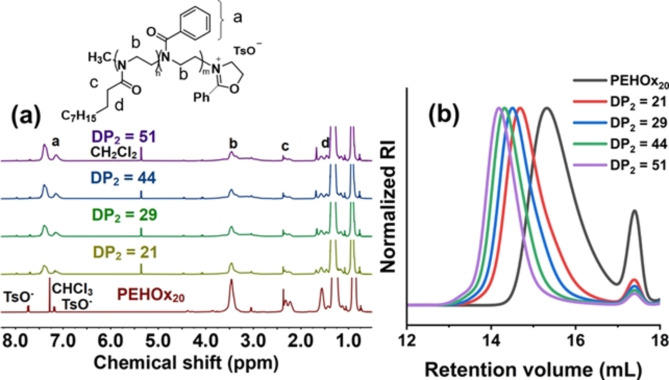
PEHOx_20_ and PEHOx_20_‐*b*‐PPhOx_m_ copolymers synthesized in *n*‐dodecane by CROPISA. (a) ^1^H NMR spectra acquired in CDCl_3_ or CD_2_C_l2_. (b) SEC traces of copolymers in DMAc eluent

^1^H NMR spectroscopy of the prepared PEHOx‐*b*‐PPhOx copolymers confirmed the targeted chemical structure and nearly complete conversion of the monomers in all the cases (Figure 2A). DP_1_ values were calculated from the signal integral areas belonging to the PEHOx units and the signal of the initiator‐originated tosylate counteranion (TsO^−^), as similarly reported in literature,[^60^, ^61^] since the terminal living 2‐oxazolinium end‐group is susceptible to hydrolysis and, therefore, unreliable for end‐group analysis. MALDI‐ToF MS analysis of the first block was used to validate this method. The degree of polymerization (DP) obtained by MALDI‐ToF MS closely matched both the DP derived from ^1^H NMR analysis and the targeted values (Figure S3). DP_2_ values were obtained by comparing the integrals of the aromatic PPhOx protons with those of the PEHOx units in the ^1^H NMR spectra.

Furthermore, SEC analysis confirmed the successful chain extension by the shift of the chromatogram peaks towards lower retention times after polymerization of the second block (Figure 2B and S12). The synthesized block copolymers containing shorter DP_1_ blocks were well defined, as demonstrated by the narrow unimodal distribution of the copolymers and their low dispersity.

Copolymers with a longer DP_1_ (>23) showed a bimodal distribution in SEC due to the occurrence of chain‐transfer reactions, which will be discussed in more detail in the section devoted to gradient CROPISA. Therefore, our further research on diblock CROPISA focused mainly on copolymers with shorter shell‐forming blocks with DP_1_≤23. The SEC‐based number‐averaged molar masses (*M*_n_) were greater than the theoretical values because of the use of PMMA calibration standards, which are not ideal for the analysis of such diblock copolymers and commonly leads to overestimation of the *M*_n_ of poly(2‐oxazoline)s.

**Figure 3:**
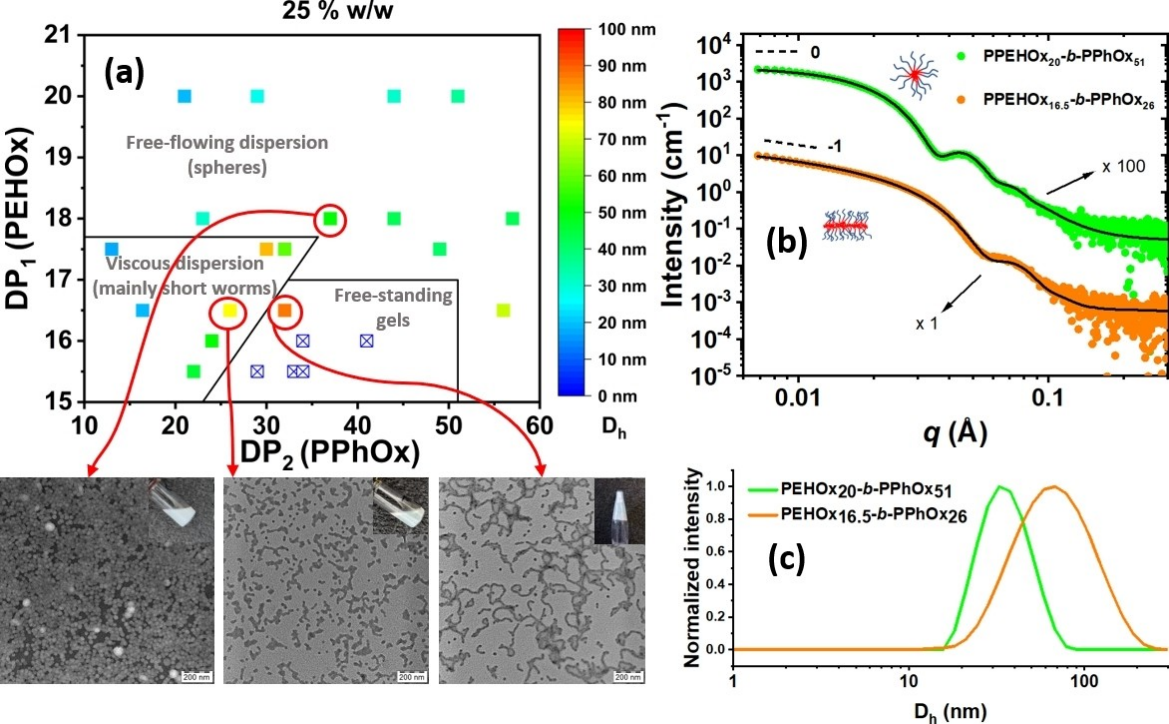
(a) Phase diagram of PEHOx_n_‐*b*‐PPhOx_m_ copolymer nanoparticles prepared by CROPISA in *n*‐dodecane at a 25 % total solid weight concentration. Representative nanoparticle morphologies were determined by TEM upon dilution with *n*‐dodecane to 1 mg mL^−1^ (left micrograph after negative staining with uranyl acetate) ) or 0.25 mg mL^−1^ in hexane (middle and right). The included pictures of the dispersions have been taken several months after synthesis, providing a visual confirmation of their stability upon storage. (b) Small‐angle X‐ray scattering (SAXS) patterns (dots) and fittings (solid lines) obtained for 1.0 % w/w dispersions of PEHOx_20_‐*b*‐PPhOx_51_ spheres and PEHOx_16.5_‐*b*‐PPhOx_26_ cylinders in *n*‐dodecane at 20 °C. Dashed lines for low q values indicate gradients of 0 and −1, which are consistent with sphere and worm morphologies, respectively (c) Respective hydrodynamic diameter distributions of nanoparticles in *n*‐dodecane measured by DLS.

**Figure 4:**
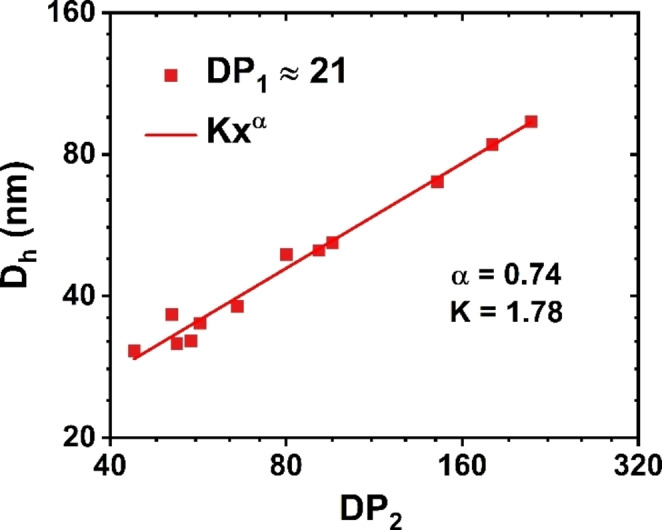
Hydrodynamic diameter (*D*_h_) vs. degree of PhOx polymerization (DP_2_) for a series of diblock PEHOx_n_‐*b*‐PPhOx_m_ copolymer nanoparticles with similar degrees of polymerization of the PEHOx block (DP_1_=20–23). The linear increase in *D*_h_ with increasing DP_2_ in the double logarithmic plot underscores the power law governing their relationship.

The nanoparticle phase diagram shows three distinct regions on the basis of the macroscopic appearance (Figure 3A). For copolymers with longer stabilizing PEHOx blocks (DP_1_≥18), the resulting dispersions appeared as low‐viscosity colloidal solutions with hydrodynamic diameters gradually increasing with increasing DP_2_. When the *D*_h_ values of the nanoparticles were plotted against DP_2_ while keeping comparable PEHOx lengths, they followed the theoretical power relationship *D*_h_=k ⋅ DP_2_^ɑ^ with ɑ=0.74 (Figure 4). This result was consistent with the adoption of a stretched conformation by the core‐forming chains.[^62^, ^63^] Furthermore, the nanoparticles in this region had a narrow size distribution (PDI<0.1 for DP_1_ >18) and proved to be stable even at relatively high PPhOx/PEHOx block ratios (Table S1). Transmission electron microscopy (TEM) of selected formulations in this region (Figure 3A and S4) revealed the presence of spherical nanoparticles with dimensions that are consistently slightly lower than the values derived by DLS. This observed difference arises because DLS reports a z‐average diameter, whereas TEM provides a number‐average diameter. Also, the loss of solvent from the shell‐forming block during the drying of the TEM samples can result in the shrinking of polymer nanoparticles.^[64]^

A second distinct region comprising polymers with shorter solvophilic PEHOx blocks (DP_1_ <18) was characterized by dispersions with substantially higher viscosities. Significant variations in *D*_h_ values along DP_2_ were accompanied by a lack of observable trends or patterns and relatively high nanoparticle size dispersities (PDI=0.07–0.37). Interestingly, similar maximal DP_1_ values (17–18) have been reported in the literature to be critical for the formation of higher nanoparticle morphologies in analogous PISA systems performed in non‐polar aliphatic solvents.[^65^, ^66^, ^67^] These systems are characterized by the use of methacrylic or acrylic monomers with long alkyl side chains or aromatic side groups. Under these conditions, equal or lower DP_1_s were reported to allow the micelles to transition into worms and vesicles. Instead, higher DP_1_ values prevented higher‐order morphology formation because of the increased steric stabilization provided by the first solvophilic block. The high viscosity of the nanoparticle solutions in this region in our work could originate from the presence of worm‐like micelles in the solution. Indeed, TEM revealed the presence of worm‐like nanoparticles in the solution, although they appeared branched and mixed with spherical nanoparticles (Figure 3A).

Increasing the core‐forming block DP_2_ in this region, in almost every instance, led to the formation of a free‐standing gel, which constituted the third phase of the diagram. The gels obtained in all but one instance were not soluble upon further dilution in *n*‐dodecane and, therefore, are reported in the diagram without an associated *D*_h_ value. Since the gels were still soluble in acetonitrile, we believe that the physical crosslinking of worm‐like micelles through their aliphatic shells prevented dissolution by the addition of *n*‐dodecane because the gel was frozen in a kinetically trapped state due to the high glass transition temperature of PPhOx (~105 °C).^[48]^ TEM analysis of the dispersible gel showed the presence of worms that appeared clustered and intertwined, confirming that gelation arises from the physical crosslinking of these worms. Again, the presence of spherical nanoparticles was also noted.

To gain a more comprehensive insight into the morphology of the nanoparticles in the viscose region, we studied selected nanoparticles via small‐angle X‐ray scattering (SAXS) and compared them with one sample from the free‐flowing region (Figure 3B). A core‐shell cylinder model provided a good fit of the experimental data for the viscous nanoparticle solution, with a calculated cylinder length of ~40 nm and diameter of ~10 nm. A core‐shell sphere model fitting performed similarly well for the free‐flowing dispersion, with a calculated sphere diameter of 36 nm, comparable with the DLS data (Figure 3C). These results suggest the presence of different nanoparticle morphologies associated with the different rheological properties of the formulations. In particular, the formation of spherical nanoparticles for DP_1_ > 18 explains the decrease in viscosity notwithstanding the longer polymer chains, while the presence of short worm‐like nanoparticles seems to be responsible for the high viscosity registered at lower DP_1_s. Notably, György and coworkers reported that relatively short worms in *n*‐dodecane can actually generate high‐viscosity dispersions and even free‐standing gels.^[68]^ Furthermore, employing the relatively high *T*_g_ core‐forming polymer blocks for PISA in non‐polar media appeared to be correlated with the lack of formation of higher nanoparticle morphologies, such as long worms and vesicles, similar to what we observed in our system. The effect of the total solids concentration on dispersions was also investigated by generating an analogous phase diagram displaying formulations synthesized at different concentrations and DP_2_s (Figure S5). As in the previous case, the same three phases were obtained with one additional phase consisting of unstable nanoparticles that precipitated during polymerization.

**Scheme 2:**
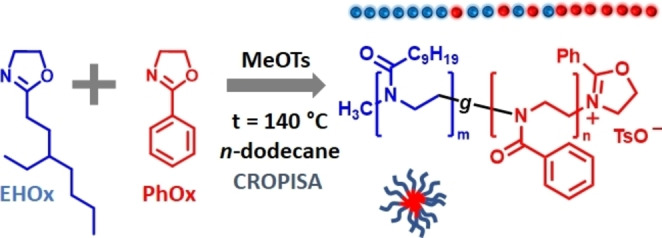
General outline of the synthesis of PEHOx_m_‐*g*‐PPhOx_n_ gradient copolymer nanoparticles via single‐step CROPISA in *n*‐dodecane.

In the next step, we developed a straightforward protocol for one‐pot, one‐step CROPISA synthesis of nanoparticles directly from monomers by gradient copolymerization of EHOx and PhOx in *n*‐dodecane (Scheme 2). Since the homopolymerization of 2‐alkyl‐2‐oxazolines has been reported to proceed significantly faster than the homopolymerization of PhOx,^[57]^ the aim was to afford analogous nanoparticle formulations by synthesizing gradient copolymers in situ via self‐assembly in *n*‐dodecane. In this way, similar nanomaterials might be obtained in a faster, more streamlined one‐step protocol.

**Figure 5:**
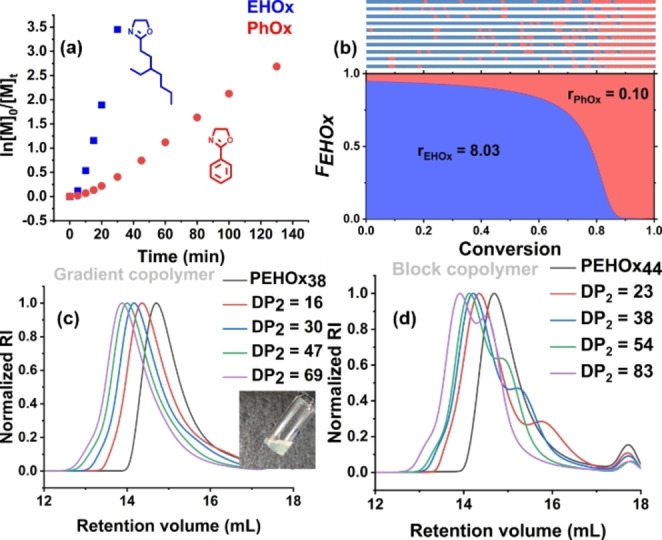
(a) Semilogarithmic conversion plot over time for the copolymerization of EHOx and PhOx in *n*‐dodecane at 140 °C, [EHOx]0:[PhOx]0:[MeOTs]0=63:27:1, initial monomer concentration of 25 wt.% (b) Visualization of the gradient copolymer microstructure via kinetic Monte Carlo simulation for 10 copolymer chains (top) and a Skeist diagram (bottom). (c) SEC traces of gradient copolymers synthesized in *n*‐dodecane at 140 °C by gradient CROPISA. (d) SEC traces of analogous diblock copolymers synthesized by a CROPISA in *n*‐dodecane at 140 °C.

The gradient copolymerization kinetics during CROPISA were monitored by GC (Figure 5A) and revealed significantly faster consumption of EHOx than of PhOx. Upon reaching approximately 85 % EHOx conversion, an acceleration of PhOx propagation was observed. DLS measurements revealed that at approximately the same time, there was a sharp increase in the hydrodynamic diameter, corresponding to self‐assembly accompanied by faster core‐forming monomer propagation, in line with the kinetic model of PISA reported in the literature (Figure S6). Notably, when the same gradient copolymerization was performed under homogeneous conditions in acetonitrile (Figure S7), the rate of polymerization of PhOx was significantly lower, indicating the partitioning of the PhOx monomer to the phase‐separated cores during CROPISA. While in *n*‐dodecane, PPhOx reached 93 % conversion after 140 min, in acetonitrile, the conversion was only 31 % after 150 min. The polymerization rate of solvophilic EHOx in acetonitrile was lower than that in *n*‐dodecane, but the difference was less marked.

The reactivity ratios of both monomers, *r*_EHOx_ = 8.03 and *r*_PhOx_ = 0.10, were calculated by fitting the kinetic data with the Meyer–Lowry equation (Figure S8), which suggested the presence of a steep compositional gradient. The reactivity ratios were employed to visualize the gradient microstructure via the Skeist diagram and to perform a kinetic Monte Carlo simulation with 10 copolymer chains (Figure 5b). Notably, the simulation highlights the slow incorporation of PhOx at an early stage of copolymerization, leading to its minor incorporation in the nanoparticle shell, while the majority of PhOx is included in the final part of the copolymers forming the core. Having confirmed the livingness of the copolymerization, the formation of a compositional gradient along the chains, and the self‐assembly of the copolymers in the nanoparticles, we focused on the preparation of well‐defined and stable nanoparticles.

Gradient CROPISA proved to be feasible for the direct synthesis of nanoparticles with EHOx blocks longer than 20 units. A shorter DP_1_ led to aggregation and precipitation of the nanoparticles. Therefore, the targeted DP_EHOx_ was set at 40, while DP_PhOx_ varied between 16 and 69, and the total solids concentration was kept constant at 25 wt.% for all formulations. Stable nanoparticles were obtained for DP_2_ values of up to 30, and DLS analyses revealed that the size of the nanoparticles reached 41 nm (Table S1). Nanoparticles with longer DP_2_ (> 30) lack colloidal stability and sediment upon cooling the reaction mixture to room temperature, presumably due to the presence of solvophobic PPhOx units in the micelle shells.

In all the cases, it was possible to prepare gradient copolymers with accelerated reaction rates compared with solution polymerization, and relatively low dispersity (*Đ* ≤ 1.41), and total DPs of up to 107 (Figure 5C). Interestingly, when copolymers with similar compositions were synthesized by the sequential block copolymer CROPISA route, a precipitate formed during polymerization, which underscores the potential of the one‐step gradient CROPISA technique. Further inspection of the SEC chromatograms of the block copolymer precipitate revealed a bimodal distribution of the molecular weights (Figure 5D). A comparison of the UV signal with the RI signal (Figure S9) clearly revealed that the low‐molecular‐weight shoulders are richer in UV‐absorbing PPhOx units. This finding indicates significant chain transfer side reactions upon polymerization of the second PhOx monomer, a phenomenon widely known in the poly(2‐oxazoline) literature.[^57^, ^69^, ^70^] In particular, inferior control over the polymerization of 2‐oxazoline monomers with long alkyl side chains has been previously reported.^[71]^ For example, Wiesbrock et al. reported the formation of a low molar mass shoulder in the SEC traces of a poly(2‐nonyl‐2‐oxazoline) chain‐extended with a poly(2‐ethyl‐2‐oxazoline) block and, to a lesser extent, with a poly(2‐phenyl‐2‐oxazoline) (PPhOx) block;^[72]^ this was explained by chain‐transfer reactions stemming from the heterogeneity of the system. In their case, the heterogeneity arose from poly(2‐nonyl‐2‐oxazoline) not being soluble at room temperature in the solvent used (acetonitrile); in our system, the formation of micelles could play a similar role.

To demonstrate a possible application of our system, we investigated the use of the nanoparticle solution obtained by CROPISA for the stabilization of water‐in‐oil Pickering emulsions. Two block copolymer formulations, PEHOx_15.5_‐*b*‐PPhOx_22_ (worms) and PEHOx_20_‐*b*‐PPhOx_101_ (spheres), with different nanoparticle morphologies but similar D_h_ values (46 and 52 nm, respectively), have been tested as emulsifying stabilizers for a 90/10 (v/v) emulsion of dodecane/water at different polymer concentrations (Figure S10). The dependence of the droplet size on the total solid weight percentage is an indication that the emulsion is stabilized by the nanoparticles and not by free polymer chains. Notably, the worm CROPISA formulation yielded smaller emulsion droplets compared to spheres, as also observed by Thompson, et al. for other types of polymer particles,^[73]^ which might be beneficial in applications like Pickering interfacial catalysis.^[74]^

## Conclusion

In this report, we described for the first time the synthesis of nanoparticles via polymerization‐induced self‐assembly (PISA) via cationic ring‐opening polymerization (CROP) of 2‐oxazolines. One‐pot cationic ring‐opening polymerization‐induced self‐assembly (CROPISA) was demonstrated to be a straightforward synthetic approach toward for preparation of stable poly(2‐(3‐ethylheptyl)‐2‐oxazoline)‐block‐poly(2‐phenyl‐2‐oxazoline) copolymer nanoparticles in *n*‐dodecane with hydrodynamic diameters up to 94 nm. The physical properties of the dispersions depend on the length of both blocks, the copolymer concentration, and the nanoparticle morphology. Within certain intervals of these parameters, the formulations shared similarities in their macroscopic behavior, and the dependence of nanoparticle size and dispersity on the degree of polymerization of the core‐forming block was observed. Based on these data, phase diagrams were generated to provide a more comprehensive picture, assist in the synthesis of nanoparticles, and highlight the robustness of the method overall. Interestingly, the kinetic data obtained for the polymerization of the second block deviated from what is commonly reported for PISA in the literature, presenting linear pseudo‐first‐order kinetics for the entire range of conversions. This unexpected behavior was determined to be the result of the formation of nanoparticles at very short lengths of the solvophobic block, as highlighted by the DLS study of the kinetic samples. Overall, the polymerization was observed to proceed in a living manner and with limited chain transfer reactions as long as the DP of the solvophilic block was kept below 23.

Finally, we developed a one‐step method for the synthesis of nanoparticles directly from monomers via the statistical copolymerization of EHOx and PhOx, which are monomers with significantly different reactivities and solvophilicities in *n*‐dodecane. Then, a statistical copolymerization of EHOx and PhOx led to formation of an amphiphilic gradient copolymer that self‐assembled in situ. From the kinetic study of the gradient copolymerization, we derived the reactivity ratios of the two monomers (*r*_EHOx_=8.03 and *r*_PhOx_=0.10), confirming the significant difference in comonomer reactivities necessary to synthesize copolymers with a sufficiently steep gradient to undergo self‐assembly.

In the future, we will explore the potential of our system for the formulation of low‐viscosity lubricants and Pickering emulsion stabilizers for which preliminary proof‐of‐concept were also reported in this work. Compared to other similar systems, our CROPISA system provides the advantage of generating nanoparticles with a high *T*_g_ core that should enhance their colloidal stability. Such nanoparticles have already been prepared employing polymethylmethacrylate as the core‐forming block. However, to the best of our knowledge, worm morphologies have consistently been challenging to achieve via PISA with PMMA as core due to the presence of kinetically trapped spheres because of the high core rigidity.^[75]^ To address this, studies have explored the incorporation of a third monomer and crosslinking to access worms with stiffer cores.^[76]^ While effective, these techniques add complexity to the synthetic protocol. Furthermore, poly(2‐oxazoline)s benefit from the increase in main chain flexibility and polarity compared to poly(meth)acrylates which might further contribute to lubrication performance.^[77]^ In addition, the nanoparticles are directly synthesized in a medium (*n*‐dodecane) that is compatible with direct dilution in mineral oil. Finally, the emerging one‐step gradient CROPISA will be further investigated as a straightforward tool for the construction of advanced nanoparticles.

## Supporting Information

The authors have cited additional references within the Supporting Information^[61]^

## Conflict of Interests

RH is a cofounder of Avroxa BV that commercializes poly(2‐oxazoline)s. The other authors have no conflicts to declare.

## Supporting information

As a service to our authors and readers, this journal provides supporting information supplied by the authors. Such materials are peer reviewed and may be re‐organized for online delivery, but are not copy‐edited or typeset. Technical support issues arising from supporting information (other than missing files) should be addressed to the authors.

Supporting Information
