# Supplementary material for: Cationic Ring‐Opening Polymerization‐Induced Self‐Assembly (CROPISA) of 2‐Oxazolines: From Block Copolymers to One‐Step Gradient Copolymer Nanoparticles
Source: Angew Chem Int Ed Engl. 2024 Dec 9;64(4):e202416106. doi: 10.1002/anie.202416106 (PMC11753605; doi:10.1002/anie.202416106)
Supplement: Supplementary file 1 — Supporting Information [file ANIE-64-e202416106-s001.pdf]

Angewandte Chemie
Eine Zeitschrift der Gesellschaft Deutscher Chemiker

Supporting Information

Cationic Ring-Opening Polymerization-Induced Self-Assembly (CROPISA) of 2-Oxazolines: From Block Copolymers to One-Step Gradient Copolymer Nanoparticles

N. Lusiani, E. Pavlova, R. Hoogenboom, O. Sedlacek**

Supplementary information

Cationic ring-opening polymerization-induced self-assembly (CROPISA) of 2-oxazolines: From block copolymers to one-step gradient copolymer nanoparticles

Niccolò Lusiani,[a] Ewa Pavlova,[b] Richard Hoogenboom[c,*] and Ondrej Sedlacek[a,*]

[a]Department of Physical and Macromolecular Chemistry, Faculty of Science, Charles University, 128 40 Prague 2, Czech Republic

E-mail: richard.hoogenboom@ugent.be, sedlacek@natur.cuni.cz

[b]Institute of Macromolecular Chemistry, v.v.i., Academy of Sciences of the Czech Republic, Heyrovsky Sq. 2, 162 06 Prague 6, Czech Republic

[c]Department of Organic and Macromolecular Chemistry, Supramolecular Chemistry Group, Centre of Macromolecular Chemistry (CMaC), Faculty of Sciences, Ghent University, Krijgslaan 281 S4, 9000 Ghent, Belgium

1. Experimental section

1.1. Materials

2-Phenyl-2-oxazoline (PhOx) was purchased from Sigma Aldrich, distilled from barium oxide, and stored under argon. 2-(3-Ethylheptyl)-2-oxazoline (EHOx) was prepared following a protocol reported in the literature for the synthesis of 2-alkyl-2-oxazolines,[46] distilled over barium oxide and stored under argon. Anhydrous acetonitrile and dodecane, supplied with molecular sieves under nitrogen atmosphere (AcroSeal by Across), were used as received. Methyl *p*-toluenesulfonate (MeOTs) was purchased from Sigma Aldrich and used as received. All other chemicals were purchased from Sigma-Aldrich unless stated otherwise.

1.2. Synthesis of PEHOx-PPhOx copolymer nanoparticles via CROPISA

A one-pot, two-step protocol was employed for the synthesis of the block copolymers (**Scheme 1**). Several batches of block copolymer nanoparticles were synthesized, with each batch characterized by the same degree of polymerization of the first block (DP_1) and total solid weight percentage (w/w %) but different degrees of polymerization of the second block (DP_2).

As a representative example, for the synthesis of the $PEHOx_{20}$-*b*-$PPhOx_y$ at 25 w/w %, MeOTs (25 μL), dodecane (1.4 mL), and EHOx (0.6 mL) were transferred under an argon atmosphere to septum-sealed microwave vials with magnetic stirrers and mixed. The vials were heated to 110 °C, and the polymerization was stopped after 1 hour by cooling down the vial to room temperature. The full conversion of EHOx was confirmed by ^1H NMR spectroscopy, and the crude polymerization mixture was split into 4 aliquots (0.4 mL) that were transferred into 4 different microwave vials under an argon atmosphere. Each microwave vial was filled with a calculated amount of PhOx (0.10-0.21 mL) according to the targeted DP_2 (30-55). Finally, a calculated volume of dodecane (0.6-1.1 mL) was added to adjust the final concentration of polymer to 25 w/w %. The polymerization of the second block was performed at 140 °C for different times (75-120 min) depending on the targeted DP_2. The polymerization mixture was cooled down to room temperature, and the conversion of PhOx was determined by ^1H NMR spectroscopy.

A similar procedure was employed for the gradient copolymer nanoparticles, with the difference that EHOx and PhOx were both added at the start in the polymerization vials, and the polymerization was performed at 140 °C in a one-step, one-pot protocol (**Scheme 2**).

1.3. Characterization of copolymers and copolymer nanoparticles

Size exclusion chromatography (SEC) was used to determine the molar masses (M_w - weight-averaged molar mass, M_n - number-averaged molar mass) and dispersity ($Đ = M_w/M_n$) of the polymers using a Malvern Omnisec system, equipped with OMNISEC RESOLVE and OMNISEC REVEAL modules, including an autosampler, a differential refractive index detector, a viscometer and a diode-array-based UV/Vis spectrometer. The separation was performed on two PLgel 5 µm mixed-D columns in a series thermostatted at 55°C in *N,N*-dimethylacetamide (DMAc) containing 50 mM of LiCl at an elution rate of 0.5 mL min^{-1}. Molar masses and dispersities were calculated against narrow-dispersity poly(methyl methacrylate) standards.

Nuclear magnetic resonance (NMR) spectra were measured on a Bruker Advance MSL 400 MHz NMR spectrometer at room temperature in $CDCl_3$ or CD_2Cl_2. All chemical shifts are expressed as ppm relative to residual solvent signals. The NMR spectra were processed using MestReNova 14.1 software.

Gas chromatography (GC) was used to monitor the CROP kinetics using a GC system (Nexis GC-2030, Shimadzu, Japan) equipped with an FID detector and a Shimadzu SH-5MS column 30 m in length and 0.32 mm in diameter. The inlet temperature was set to 250 °C with a 25:1 split ratio, and hydrogen was used as a carrier gas at a 1.8 mL min^{-1} flow rate.

Dynamic light scattering (DLS) was used to determine the hydrodynamic diameter of the copolymer nanoparticles using a ZEN3600 Zetasizer Nano-ZS analyzer (Malvern Instruments, UK). Each final polymerization crude sample was diluted in dodecane to a final concentration of 5 mg mL^{-1}. The apparent hydrodynamic diameter of the nanoparticles (D_h) and their polydispersity index (PDI) were determined at a scattering angle of $\theta = 173°$. The data were analyzed using the DTS (Nano) program.

Small-Angle X-ray Scattering (SAXS) experiments were performed at the BIOCEV center (Vestec, Czech Republic) with a SAXSpoint 2.0 instrument (Anton Paar, Graz, Austria) equipped with an Excillum MetalJet C2 X-ray source and with an Eiger R 1M detector (Dectris, Baden-Daettwil, Switzerland). The active area of the detector and the pixel size were 79.9 mm × 77.2 mm and 75 µm × 75 µm, respectively at detector distance 776.8mm, the X-ray source wavelength was

λ = 0.1348 nm. Measurements were conducted at 20 °C in a 1 mm quartz capillary, at a q vector range from 0.047 to 4.4 nm^{-1}, where $q = 4\pi.\sin\theta/\lambda$ is the length of the scattering vector and θ is one-half of the scattering angle. Data were collected for 30 min (60 frames by 30s). During the data reduction, the intensities were normalized to sample transmission and primary beam fluctuation, which was monitored using semi-transparent beamstop. SAXS curves were obtained by subtracting the solvent intensities from those for the sample. Data were modelled using the SasView 5.0.6 package.

Transmission electron microscopy (TEM) – this method was used to observe the morphology of copolymer nanoparticles. The measurements were performed in bright field mode using a Tecnai G2 Spirit Twin 120 kV TEM (FEI, Czech Republic) microscope. The nanoparticles were diluted in either hexane (DP1 < 18) or dodecane (DP1 > 18) to a concentration of 0.25 or 1 mg mL^{-1}, respectively, and were dropped on a copper TEM grid coated with a thin electron transparent carbon film. After 2 min, the excess solution was removed by touching the bottom of the grids with filtering paper to minimize oversaturation during the drying process. Dilution in hexane led to faster solvent evaporation and prevented the coalescence of worm-like nanoparticles. In this case, the particles were visible even without negative staining with uranyl acetate. Dilution by hexane instead of dodecane did not result in a significant change in nanoparticle DLS results (Figure S11). Additionally, the nanoparticles diluted in dodecane were negatively stained with uranyl acetate (2 wt.%) solution dropped on the dried nanoparticles and removed after 30 s, as described above). The samples were left to dry completely at room temperature and then observed on the microscope.

Matrix-assisted laser desorption/ionization-time of flight mass spectrometry (MALDI-TOF MS) was performed on a Microflex LT MALDI-TOF MS (Bruker Daltonics) mass spectrometer. All mass spectra were recorded at a 20 kV accelerating potential in positive ion and reflection modes (matrix: 2-[3-(4-tert-butylphenyl)-2-methyl-2-propenylidene] malononitrile (DCTB)). Samples were applied to the MALDI plate using the dried drop method. All measurements were calibrated using poly(ethylene glycol) monomethyl ether (mPEG, Mn = 2000 Da).

Preparation of Pickering emulsions – 20 μL of Nile Red dispersion in dodecane (5 mg/mL) and distilled water were added to copolymer dispersions diluted in *n*-dodecane. The mixture was homogenized for 2 min at 20 °C using an IKA Ultra-Turrax T-18 homogenizer with a 10 mm

dispersing tool operating at 12 000 rpm. The water/dodecane volume ratio was kept at 10/90 and the copolymer weight concentration in the mixture was changed between 2.00-0.25% w/w by progressive dilution.

Optical microscopy – Optical microscopy images of Pickering emulsion droplets were recorded using a Motic BA210 digital biological microscope equipped with a built-in camera and analyzed using Motic Images Plus 2.0 ML software in epifluorescent mode.

Kinetic Monte Carlo simulation code

The following simplified VBA code for visualization of repeating distribution units in copolymers was used as a macro in Microsoft Excel software. The simulation has been performed for 10 chains on ([EHOx]:[PhOx]:[MeOTs] = 63:27:1 statistical copolymerization with reactivity ratios r_{EHOx} = 8.03, r_{PhOx} = 0.10

```
Sub CopolymerizationSimulation()
    Dim rA As Double
    Dim rB As Double
    rA = 2.07 ' Reactivity ratio of monomer A
    rB = 0.16 ' Reactivity ratio of monomer B

    Dim amountA As Double
    Dim amountB As Double
    amountA = 50
    amountB = 200

    Dim chainLength As Integer
    chainLength = 250

    Dim numSimulations As Integer
    numSimulations = 100

    Dim ws As Worksheet
    Set ws = ThisWorkbook.Sheets("Sheet1")
    ws.Cells.Clear

    Randomize

    Dim sim As Integer, unit As Integer
    Dim currentAmountA As Double, currentAmountB As Double
    Dim totalAmount As Double, moleFractionA As Double, moleFractionB As
Double
    Dim randNum As Double, probA As Double, probB As Double
```

```
    Dim lastMonomer As String

    For sim = 1 To numSimulations
        currentAmountA = amountA
        currentAmountB = amountB
        lastMonomer = "A"

        For unit = 1 To chainLength
            totalAmount = currentAmountA + currentAmountB
            moleFractionA = currentAmountA / totalAmount
            moleFractionB = currentAmountB / totalAmount

            randNum = Rnd

            If lastMonomer = "A" Then
                probA = (rA * moleFractionA) / (rA * moleFractionA +
moleFractionB)
                probB = 1 - probA
            Else
                probB = (rB * moleFractionB) / (rB * moleFractionB +
moleFractionA)
                probA = 1 - probB
            End If

            If randNum < probA And currentAmountA > 0 Then
                ws.Cells(sim, unit).Value = "A"
                lastMonomer = "A"
                currentAmountA = currentAmountA - 1
            ElseIf currentAmountB > 0 Then
                ws.Cells(sim, unit).Value = "B"
                lastMonomer = "B"
                currentAmountB = currentAmountB - 1
            End If
        Next unit
    Next sim
End Sub
```

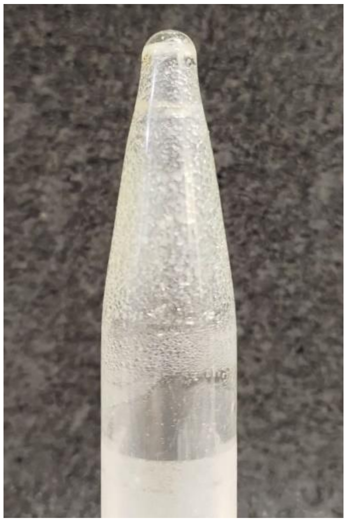

Figure S1. PPhOx precipitated on the walls of the reaction vial from the reaction crude of the homopolymerization in *n*-dodecane.

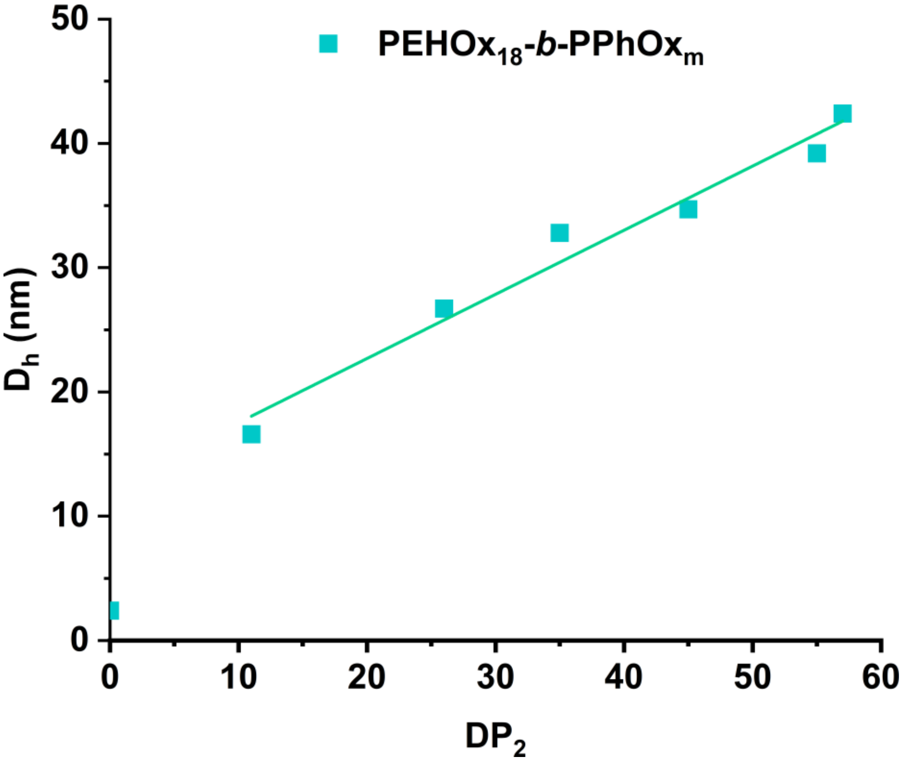

Figure S2. Hydrodynamic diameters as derived from DLS measurements for PEHOx$_{18}$-*b*-PPhOx$_{60}$ block copolymer at different conversions of the PhOx block in dodecane.

Table S1. Hydrodynamic diameters (D_h) and polydispersities (PDI) measured by DLS for different copolymer compositions, architectures and total solid weight concentrations.

Polymer	Concentration (w/w %)	D_h (nm)	PDI
$PEHOx_{17.5}$-***b***-$PPhOx_{27}$	**15**	40.2	0.156
$PEHOx_{17.5}$-***b***-$PPhOx_{32}$	**15**	90.5	0.286
$PEHOx_{17.5}$-***b***-$PPhOx_{40}$	**15**	54.2	0.088
$PEHOx_{17.5}$-***b***-$PPhOx_{45}$	**20**	NS	NS
$PEHOx_{17.5}$-***b***-$PPhOx_{52}$	**20**	NS	NS
$PEHOx_{17.5}$-***b***-$PPhOx_{70}$	**20**	72.1	0.084
$PEHOx_{15.5}$-***b***-$PPhOx_{22}$	**25**	45.9	0.194
$PEHOx_{15.5}$-***b***-$PPhOx_{29}$	**25**	NS	NS
$PEHOx_{15.5}$-***b***-$PPhOx_{33}$	**25**	NS	NS
$PEHOx_{15.5}$-***b***-$PPhOx_{34}$	**25**	NS	NS
$PEHOx_{16}$-***b***-$PPhOx_{24}$	**25**	50.9	0.249
$PEHOx_{16}$-***b***-$PPhOx_{34}$	**25**	NS	NS
$PEHOx_{16}$-***b***-$PPhOx_{41}$	**25**	NS	NS
$PEHOx_{16.5}$-***b***-$PPhOx_{16}$	**25**	19.0	0.070
$PEHOx_{16.5}$-***b***-$PPhOx_{26}$	**25**	73.9	0.245
$PEHOx_{16.5}$-***b***-$PPhOx_{32}$	**25**	87.7	0.369
$PEHOx_{16.5}$-***b***-$PPhOx_{56}$	**25**	69.6	0.113
$PEHOx_{17.5}$-***b***-$PPhOx_{13}$	**25**	18.1	0.117
$PEHOx_{17.5}$-***b***-$PPhOx_{30}$	**25**	81.2	0.247
$PEHOx_{17.5}$-***b***-$PPhOx_{32}$	**25**	58.9	0.152
$PEHOx_{17.5}$-***b***-$PPhOx_{49}$	**25**	31.5	0.022
$PEHOx_{18}$-***b***-$PPhOx_{23}$	**25**	30.9	0.256
$PEHOx_{18}$-***b***-$PPhOx_{37}$	**25**	50.0	0.166
$PEHOx_{18}$-***b***-$PPhOx_{44}$	**25**	42.4	0.114
$PEHOx_{18}$-***b***-$PPhOx_{57}$	**25**	42.4	0.065
$PEHOx_{20}$-***b***-$PPhOx_{21}$	**25**	18.9	0.056
$PEHOx_{20}$-***b***-$PPhOx_{29}$	**25**	28.1	0.078
$PEHOx_{20}$-***b***-$PPhOx_{44}$	**25**	30.6	0.065
$PEHOx_{20}$-***b***-$PPhOx_{51}$	**25**	36.6	0.099
$PEHOx_{20}$-***b***-$PPhOx_{85}$	**25**	48.6	0.055
$PEHOx_{20}$-***b***-$PPhOx_{96}$	**25**	49.8	0.056
$PEHOx_{20}$-***b***-$PPhOx_{101}$	**25**	51.6	0.057
$PEHOx_{20}$-***b***-$PPhOx_{153}$	**25**	69.5	0.050
$PEHOx_{22.5}$-***b***-$PPhOx_{53}$	**25**	31.7	0.058
$PEHOx_{22.5}$-***b***-$PPhOx_{56}$	**25**	32.1	0.055
$PEHOx_{22.5}$-***b***-$PPhOx_{58}$	**25**	35.0	0.052
$PEHOx_{22.5}$-***b***-$PPhOx_{68}$	**25**	38.0	0.058
$PEHOx_{22.5}$-***b***-$PPhOx_{180}$	**25**	83.8	0.040
$PEHOx_{22.5}$-***b***-$PPhOx_{210}$	**25**	93.8	0.094
$PEHOx_{17.5}$-***b***-$PPhOx_{15}$	**40**	16.4	0.083
$PEHOx_{17.5}$-***b***-$PPhOx_{24}$	**40**	75.8	0.335

PEHOx$_{17.5}$-***b***-PPhOx$_{39}$	**40**	NS	NS
PEHOx$_{17.5}$-***b***-PPhOx$_{47}$	**40**	NS	NS
PEHOx$_{38}$-***g***-PPhOx$_{16}$	**25**	17.3	0.126
PEHOx$_{38}$-***g***-PPhOx$_{30}$	**25**	40.5	0.198
PEHOx$_{38}$-***g***-PPhOx$_{47}$	**25**	NS	NS
PEHOx$_{38}$-***g***-PPhOx$_{69}$	**25**	NS	NS

NS: sample not soluble in dodecane

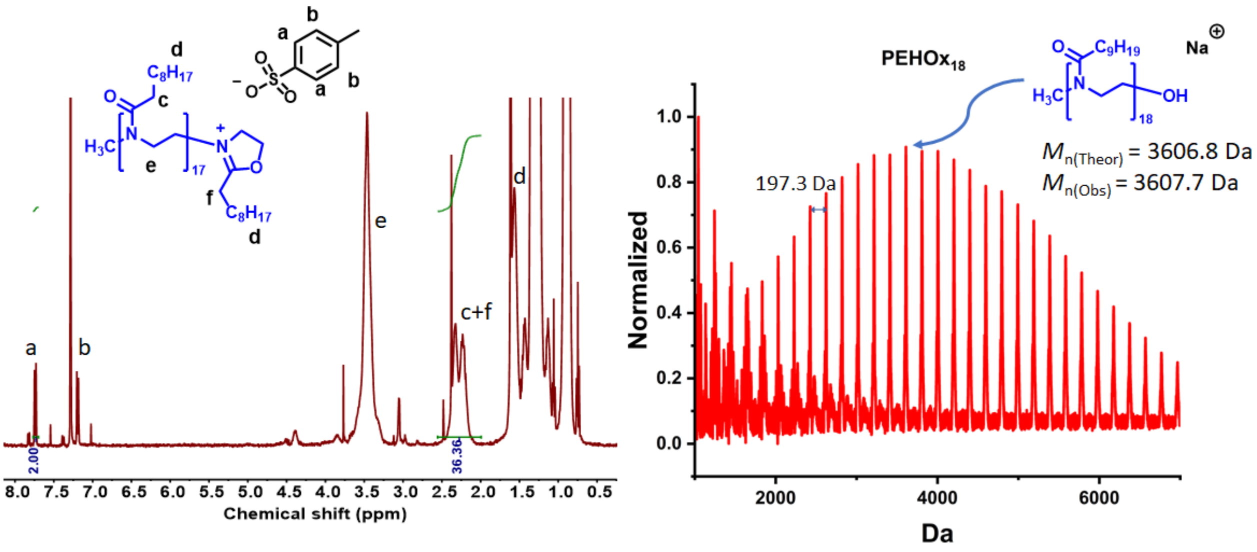

Figure S3. PEHOx$_{18}$ synthesized in dodecane. ^{1}H NMR spectrum acquired in $CDCl_3$ (left). MALDI-ToF spectrum of the water-terminated polymer (right).

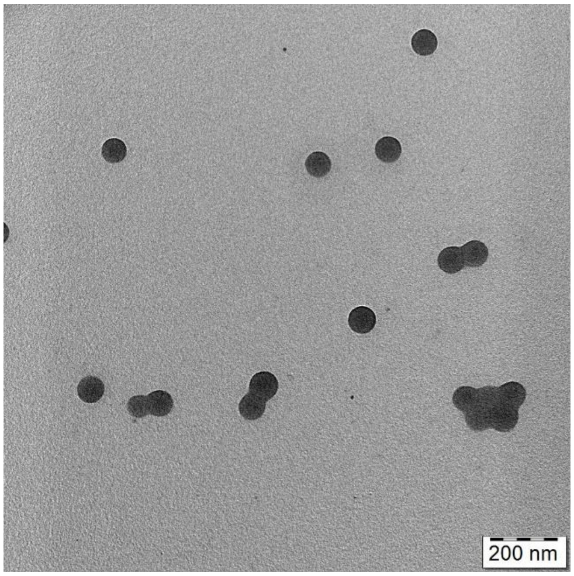

Figure S4. TEM micrographs of PEHOx$_{22.5}$-*b*-PPhOx$_{210}$ nanoparticles synthesized at 25 % w/w (diluted to 1 mg mL^{-1} in dodecane).

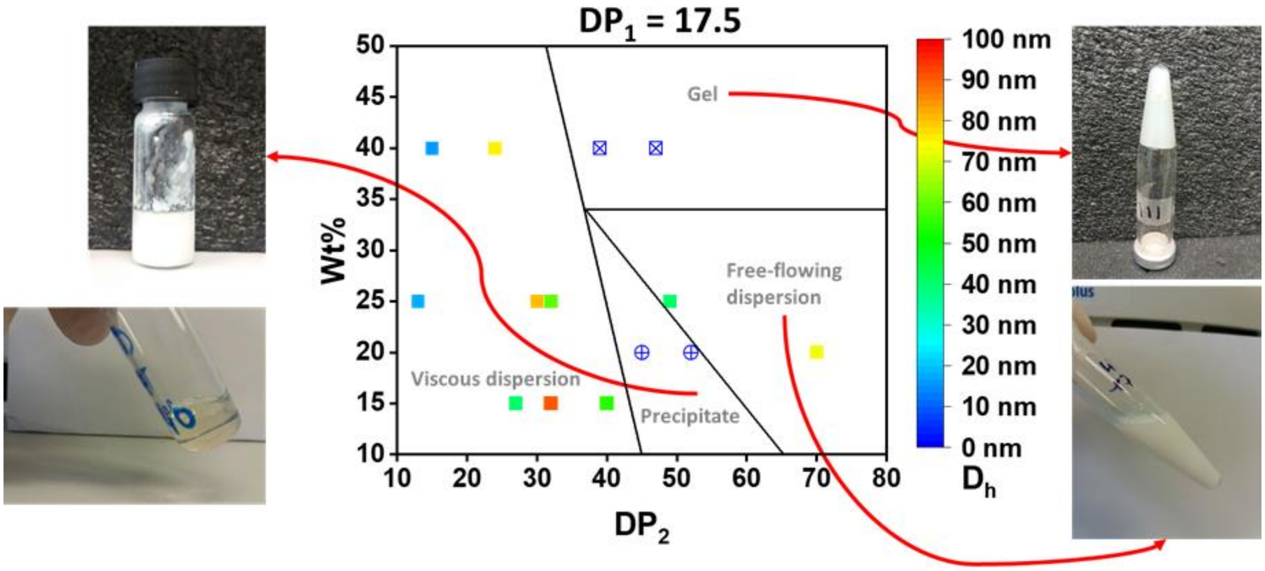

Figure S5. Phase diagram of PEHOx$_{17.5}$-*b*-PPhOx$_{m}$ copolymer nanoparticles prepared by CROPISA in dodecane at different total solid weight concentrations. A one-pot protocol was employed to synthesize all the block copolymers.

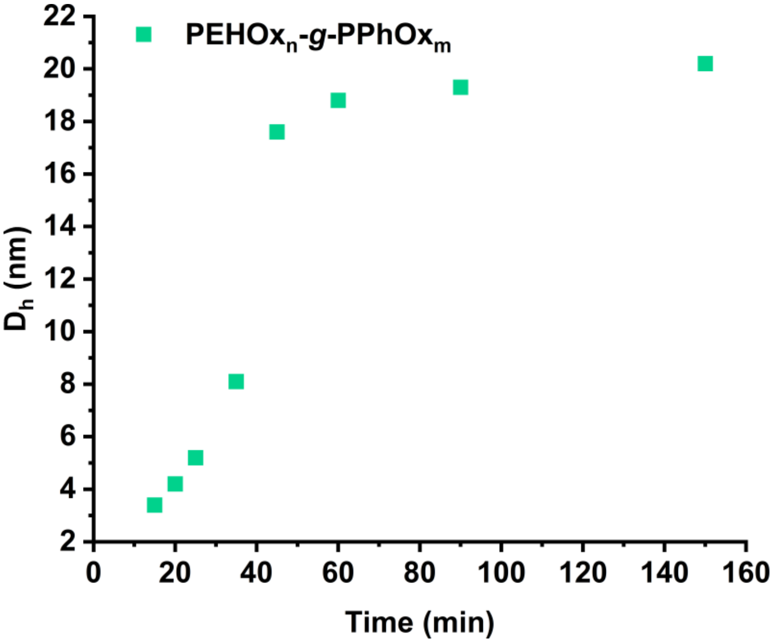

Figure S6. Hydrodynamic diameters as derived from DLS measurements for $PEHOx_{63}$-*b*-$PPhOx_{27}$ gradient copolymer synthesis at different polymerization times in dodecane.

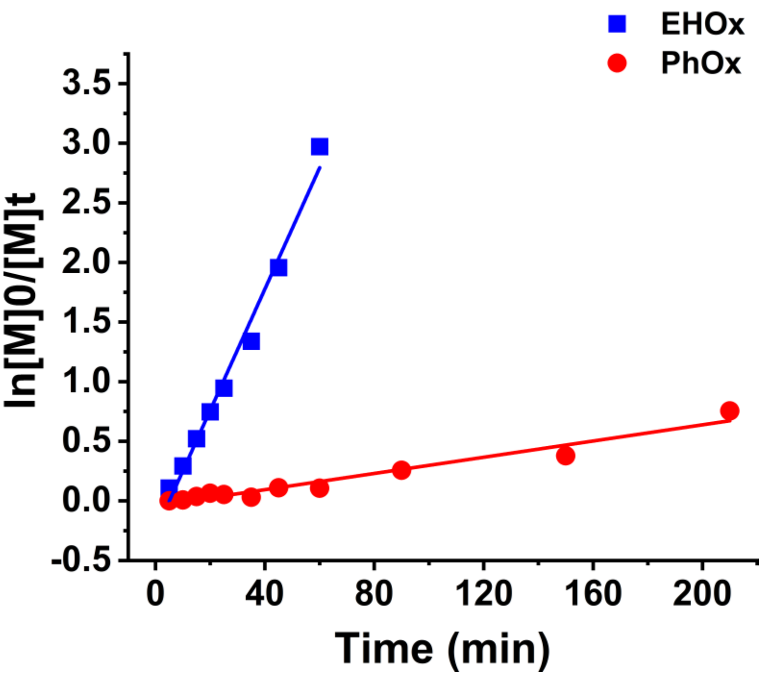

Figure S7. Semilogarithmic plot of conversion over time for the copolymerization of EHOx and PhOx in acetonitrile at 140 °C, [EHOx]:[PhOx]:[MeOTs] = 52:42:1, monomer concentration 26 wt.%

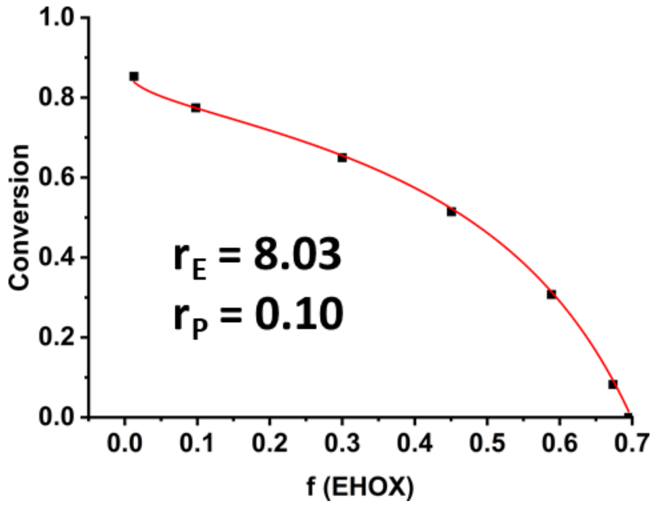

Figure S8. Meyer-Lowry plot of the copolymerization of EHOx and PhOx in dodecane at 140 °C, [EHOx]:[PhOx]:[MeOTs] = 63:27:1, monomer concentration 25 wt.% The reactivity ratios for EHOx (r_E) and PhOx (r_P) were derived as parameters from the fitting function.

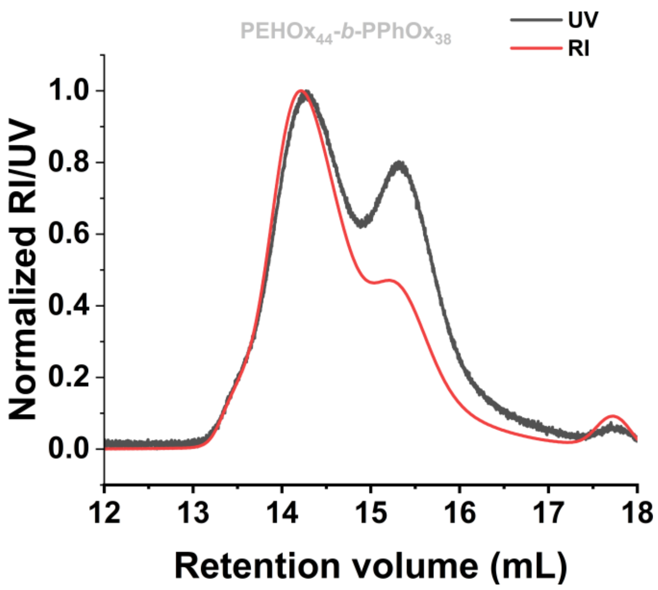

Figure S9. RI and UV SEC traces of PEHOx44-b-PPhOx38 copolymer synthesized in dodecane by a PISA protocol at 140 °C (second block). The higher content of the UV-absorbing PPhOx monomer in the low molecular weight shoulder is highlighted by the comparison with the IR trace.

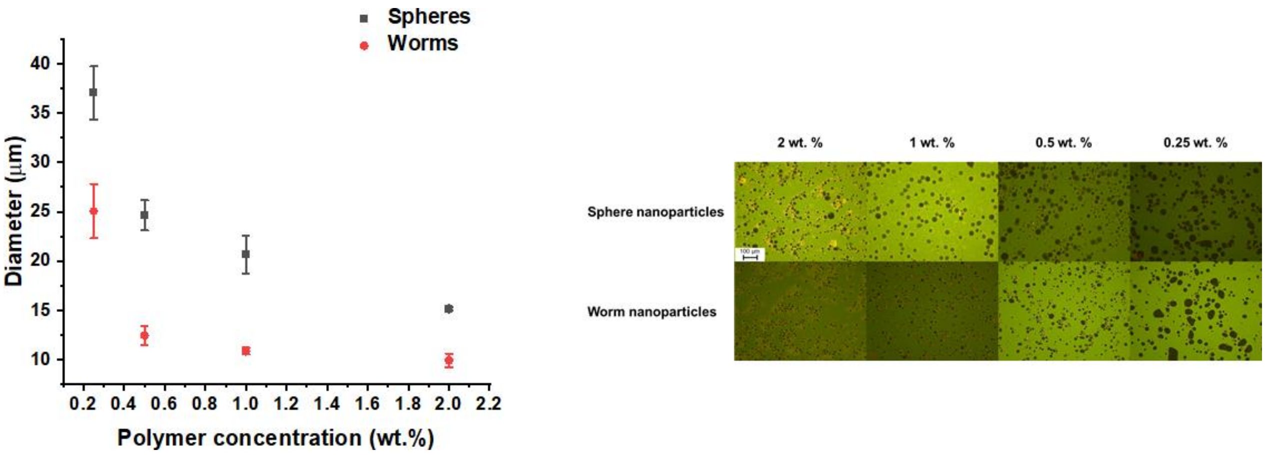

Figure S10. Mean droplet diameters *versus* copolymer concentration (left) and representative optical microscope images (right) for emulsions stabilized by $PEHOx_{15.5}$-*b*-$PPhOx_{22}$ (worms) and $PEHOx_{20}$-*b*-$PPhOx_{101}$ (spheres).

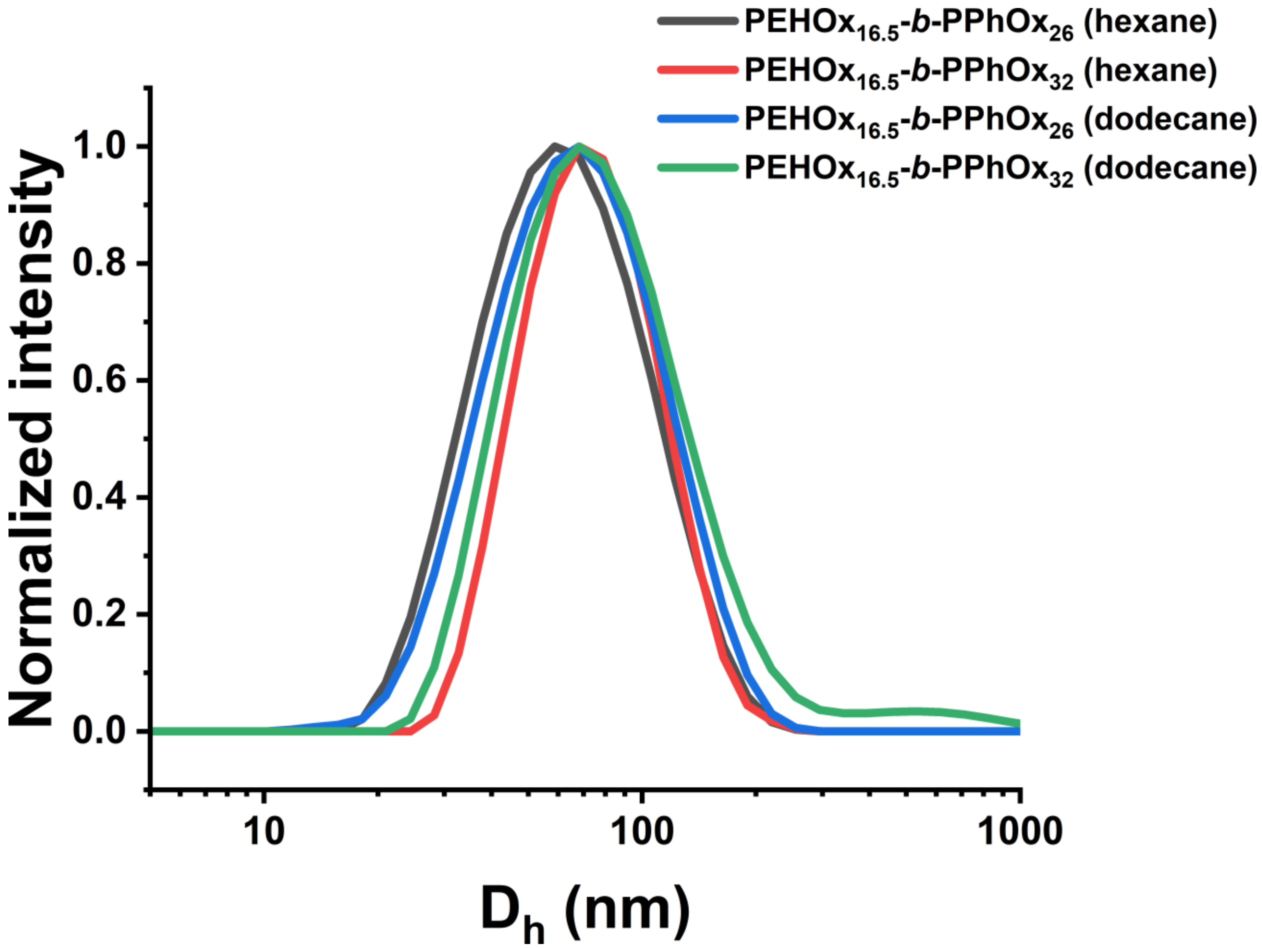

Figure S11. Hydrodynamic diameter distributions of $PEHOx_{16.5}$-*b*-$PPhOx_{26}$ and $PEHOx_{16.5}$-*b*-$PPhOx_{32}$ nanoparticles diluted in *n*-dodecane and hexane measured by DLS.

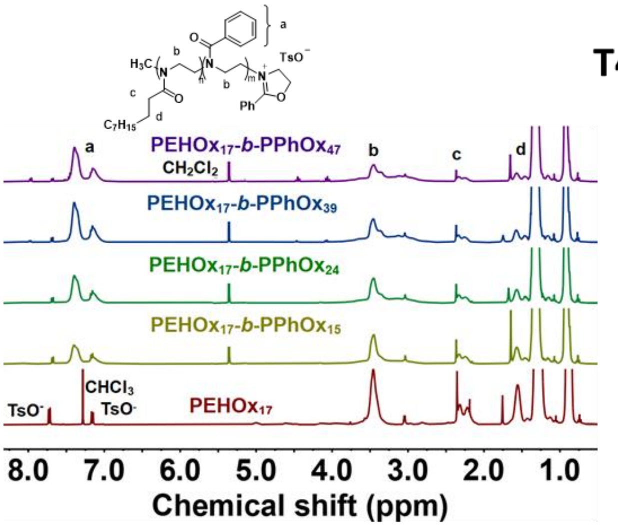
PEHOx17-b-PPhOx47
CH2Cl2
PEHOx17-b-PPhOx39
PEHOx17-b-PPhOx24
PEHOx17-b-PPhOx15
CHCl3
TsO-
PEHOx17
8.0 7.0 6.0 5.0 4.0 3.0 2.0 1.0
Chemical shift (ppm)

T4

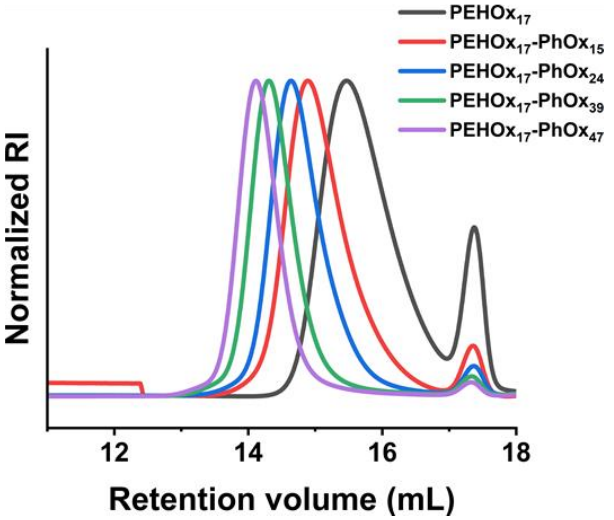
PEHOx17
PEHOx17-PhOx15
PEHOx17-PhOx24
PEHOx17-PhOx39
PEHOx17-PhOx47
Normalized RI
12 14 16 18
Retention volume (mL)

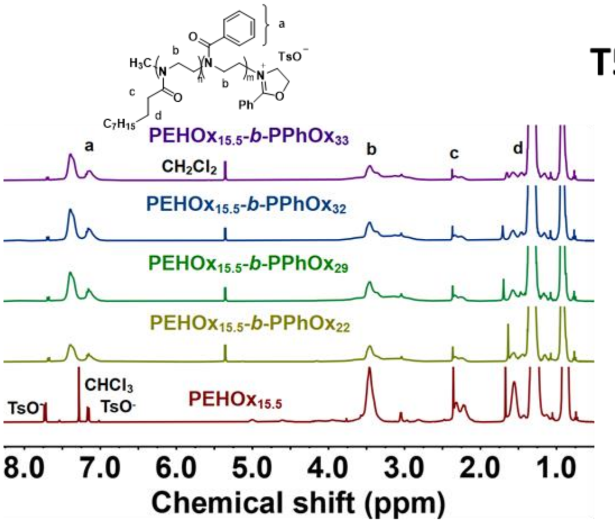
PEHOx15.5-b-PPhOx33
CH2Cl2
PEHOx15.5-b-PPhOx32
PEHOx15.5-b-PPhOx29
PEHOx15.5-b-PPhOx22
CHCl3
TsO-
PEHOx15.5
8.0 7.0 6.0 5.0 4.0 3.0 2.0 1.0
Chemical shift (ppm)

T5

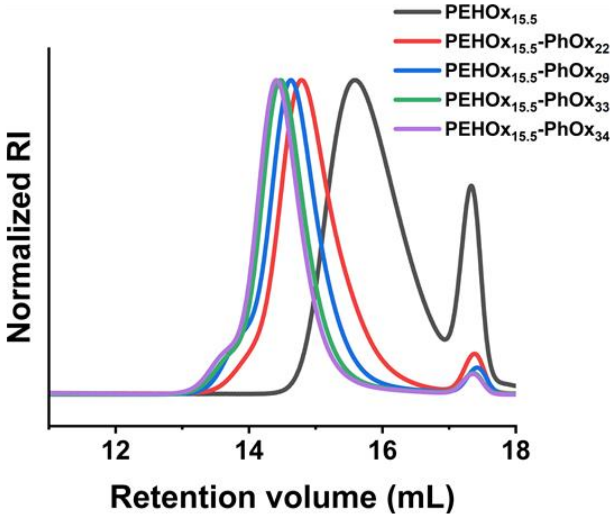
PEHOx15.5
PEHOx15.5-PhOx22
PEHOx15.5-PhOx29
PEHOx15.5-PhOx33
PEHOx15.5-PhOx34
Normalized RI
12 14 16 18
Retention volume (mL)

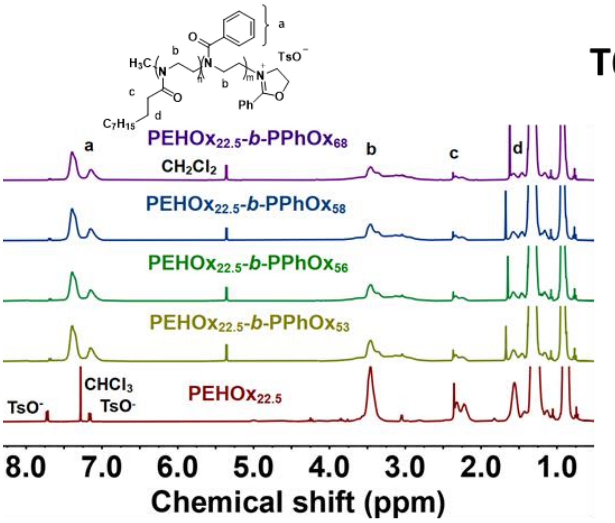
PEHOx22.5-b-PPhOx68
CH2Cl2
PEHOx22.5-b-PPhOx58
PEHOx22.5-b-PPhOx56
PEHOx22.5-b-PPhOx53
CHCl3
TsO-
PEHOx22.5
8.0 7.0 6.0 5.0 4.0 3.0 2.0 1.0
Chemical shift (ppm)

T6

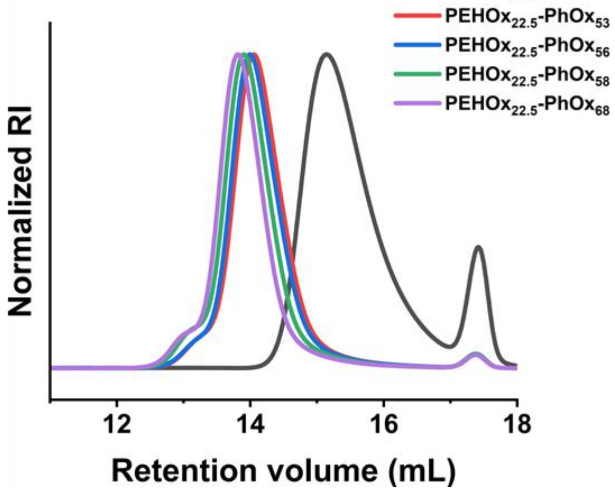
PEHOx22.5
PEHOx22.5-PhOx53
PEHOx22.5-PhOx56
PEHOx22.5-PhOx58
PEHOx22.5-PhOx68
Normalized RI
12 14 16 18
Retention volume (mL)

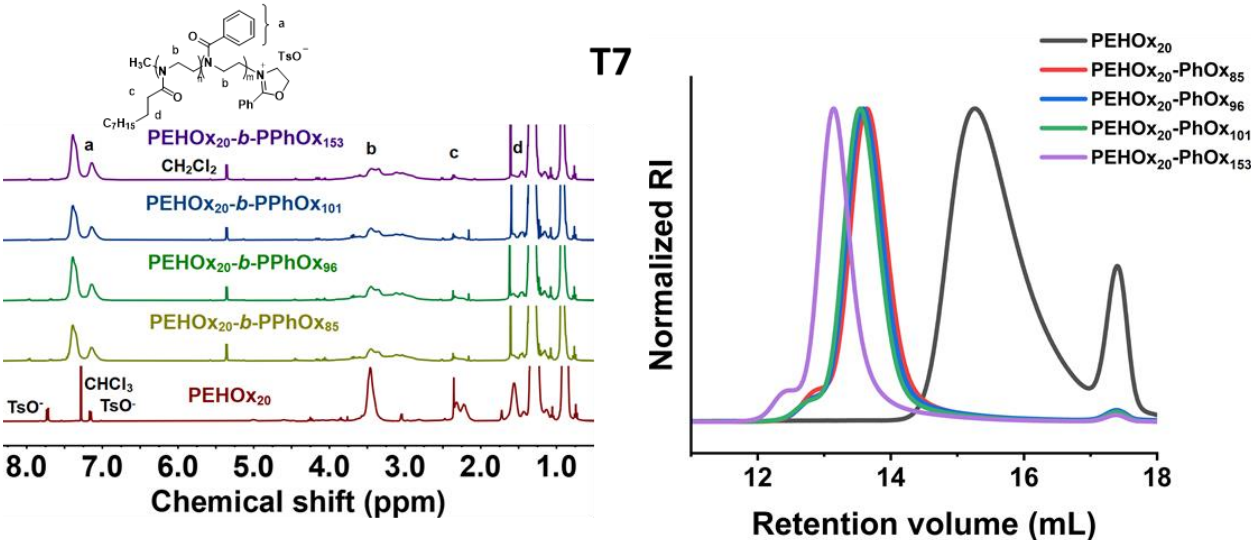

T7
PEHOx20-b-PPhOx153
PEHOx20-b-PPhOx101
PEHOx20-b-PPhOx96
PEHOx20-b-PPhOx85
PEHOx20
a
b
c
d
CH2Cl2
CHCl3
TsO-
Chemical shift (ppm)
PEHOx20
PEHOx20-PhOx85
PEHOx20-PhOx96
PEHOx20-PhOx101
PEHOx20-PhOx153
Normalized RI
Retention volume (mL)

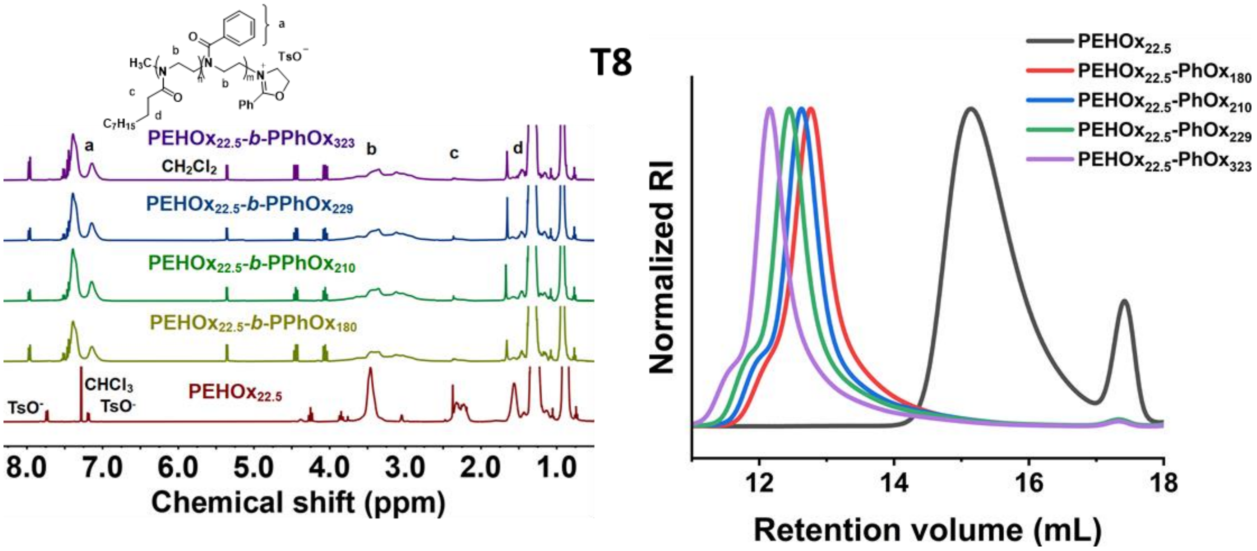

T8
PEHOx22.5-b-PPhOx323
PEHOx22.5-b-PPhOx229
PEHOx22.5-b-PPhOx210
PEHOx22.5-b-PPhOx180
PEHOx22.5
a
b
c
d
CH2Cl2
CHCl3
TsO-
Chemical shift (ppm)
PEHOx22.5
PEHOx22.5-PhOx180
PEHOx22.5-PhOx210
PEHOx22.5-PhOx229
PEHOx22.5-PhOx323
Normalized RI
Retention volume (mL)

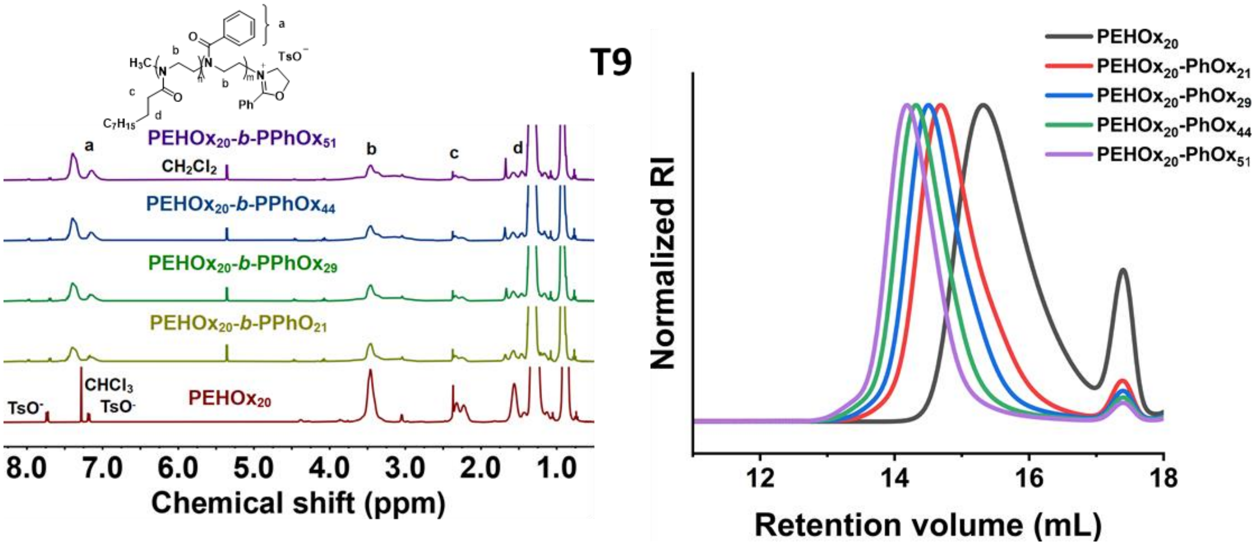

T9
PEHOx20-b-PPhOx51
PEHOx20-b-PPhOx44
PEHOx20-b-PPhOx29
PEHOx20-b-PPhO21
PEHOx20
a
b
c
d
CH2Cl2
CHCl3
TsO-
Chemical shift (ppm)
PEHOx20
PEHOx20-PhOx21
PEHOx20-PhOx29
PEHOx20-PhOx44
PEHOx20-PhOx51
Normalized RI
Retention volume (mL)

T10

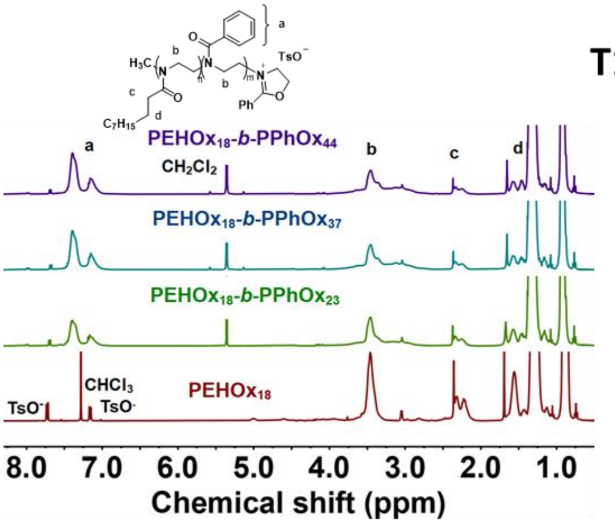

PEHOx18-b-PPhOx44
CH2Cl2
PEHOx18-b-PPhOx37
PEHOx18-b-PPhOx23
CHCl3
TsO-
PEHOx18
TsO-
Chemical shift (ppm)

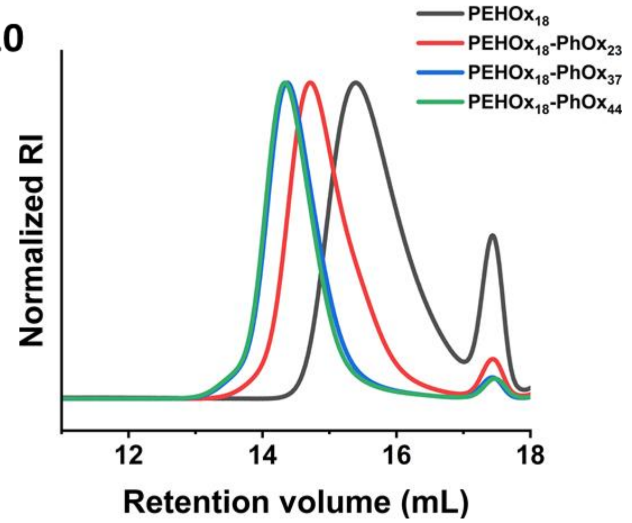

PEHOx18
PEHOx18-PhOx23
PEHOx18-PhOx37
PEHOx18-PhOx44
Normalized RI
Retention volume (mL)

T11

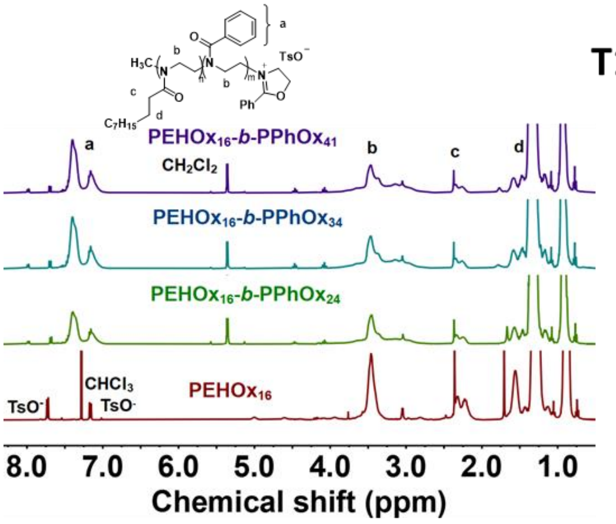

PEHOx16-b-PPhOx41
CH2Cl2
PEHOx16-b-PPhOx34
PEHOx16-b-PPhOx24
CHCl3
TsO-
PEHOx16
TsO-
Chemical shift (ppm)

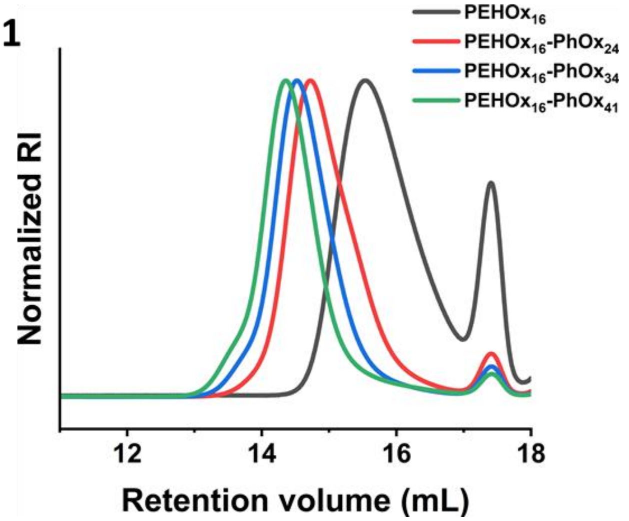

PEHOx16
PEHOx16-PhOx24
PEHOx16-PhOx34
PEHOx16-PhOx41
Normalized RI
Retention volume (mL)

T13

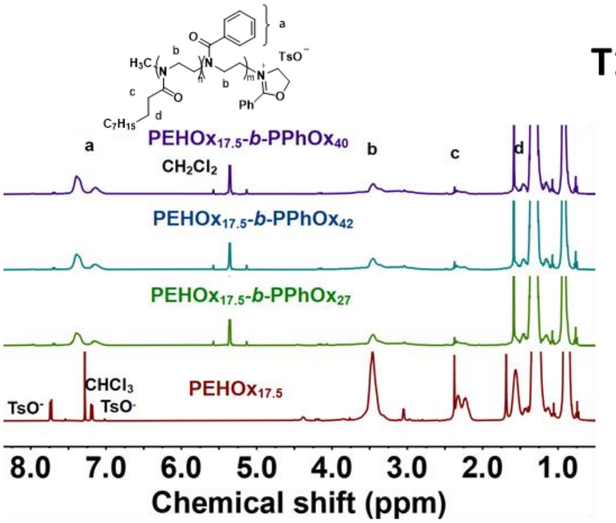

PEHOx17.5-b-PPhOx40
CH2Cl2
PEHOx17.5-b-PPhOx42
PEHOx17.5-b-PPhOx27
CHCl3
TsO-
PEHOx17.5
TsO-
Chemical shift (ppm)

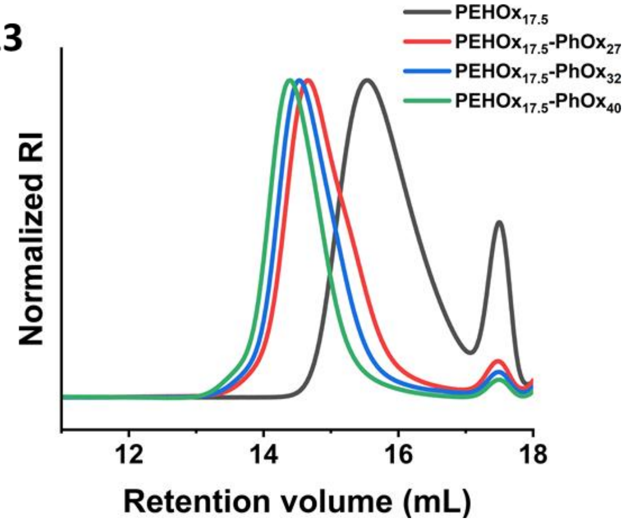

PEHOx17.5
PEHOx17.5-PhOx27
PEHOx17.5-PhOx32
PEHOx17.5-PhOx40
Normalized RI
Retention volume (mL)

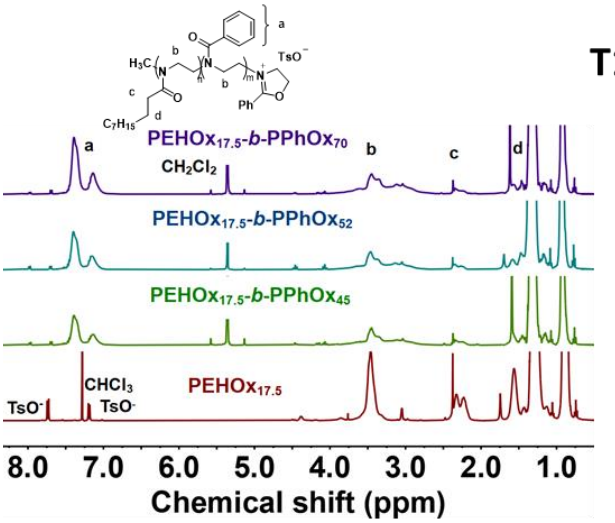

PEHOx17.5-b-PPhOx70
CH2Cl2
PEHOx17.5-b-PPhOx52
PEHOx17.5-b-PPhOx45
CHCl3
TsO-
PEHOx17.5
a
b
c
d
8.0 7.0 6.0 5.0 4.0 3.0 2.0 1.0
Chemical shift (ppm)

T14

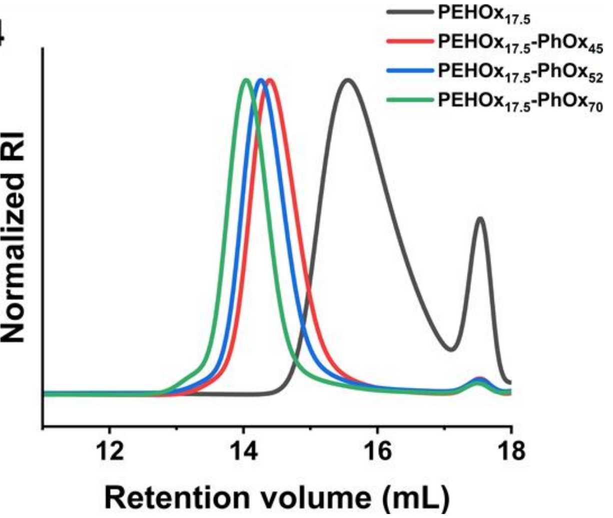

PEHOx17.5
PEHOx17.5-PhOx45
PEHOx17.5-PhOx52
PEHOx17.5-PhOx70
Normalized RI
12 14 16 18
Retention volume (mL)

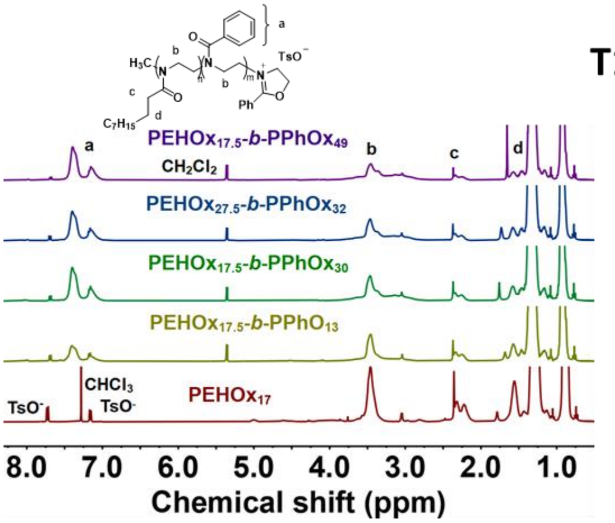

PEHOx17.5-b-PPhOx49
CH2Cl2
PEHOx27.5-b-PPhOx32
PEHOx17.5-b-PPhOx30
PEHOx17.5-b-PPhO13
CHCl3
TsO-
PEHOx17
a
b
c
d
8.0 7.0 6.0 5.0 4.0 3.0 2.0 1.0
Chemical shift (ppm)

T15

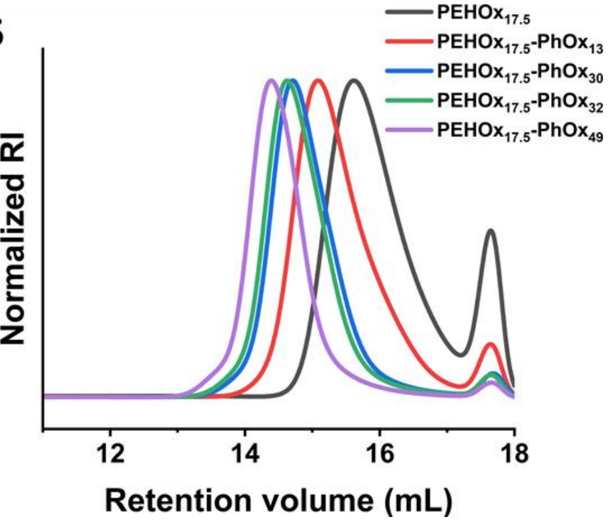

PEHOx17.5
PEHOx17.5-PhOx13
PEHOx17.5-PhOx30
PEHOx17.5-PhOx32
PEHOx17.5-PhOx49
Normalized RI
12 14 16 18
Retention volume (mL)

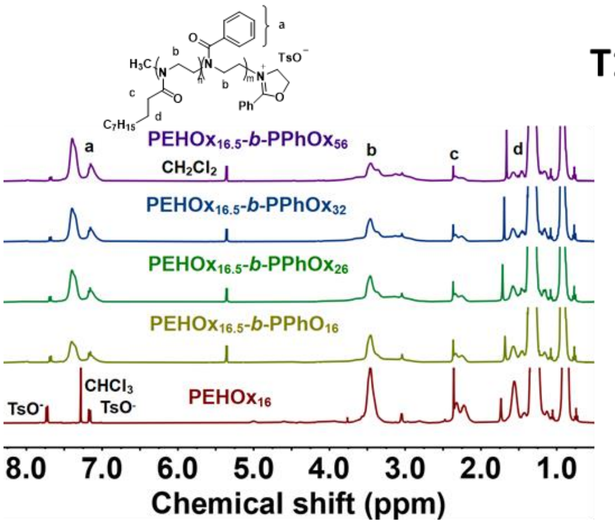

PEHOx16.5-b-PPhOx56
CH2Cl2
PEHOx16.5-b-PPhOx32
PEHOx16.5-b-PPhOx26
PEHOx16.5-b-PPhO16
CHCl3
TsO-
PEHOx16
a
b
c
d
8.0 7.0 6.0 5.0 4.0 3.0 2.0 1.0
Chemical shift (ppm)

T16

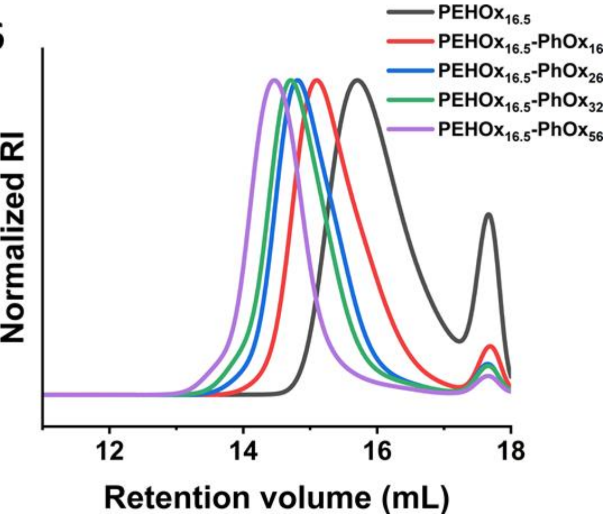

PEHOx16.5
PEHOx16.5-PhOx16
PEHOx16.5-PhOx26
PEHOx16.5-PhOx32
PEHOx16.5-PhOx56
Normalized RI
12 14 16 18
Retention volume (mL)

Figure S12. $PEHOx_n$ and $PEHOx_n$-*b*-$PPhOx_m$ copolymers synthesized in dodecane by CROPISA. ^{1}H NMR spectra acquired in $CDCl_3$ or CD_2Cl_2 (left). SEC traces of copolymers in DMAc eluent (right).
